# Design and Synthesis of Brain Penetrant Glycopeptide Analogues of PACAP With Neuroprotective Potential for Traumatic Brain Injury and Parkinsonism

**DOI:** 10.3389/fddsv.2021.818003

**Published:** 2022-01-14

**Authors:** Christopher R. Apostol, Kelsey Bernard, Parthasaradhireddy Tanguturi, Gabriella Molnar, Mitchell J. Bartlett, Lajos Szabò, Chenxi Liu, J. Bryce Ortiz, Maha Saber, Katherine R. Giordano, Tabitha R. F. Green, James Melvin, Helena W. Morrison, Lalitha Madhavan, Rachel K. Rowe, John M. Streicher, Michael L. Heien, Torsten Falk, Robin Polt

**Affiliations:** 1 Department of Chemistry and Biochemistry, BIO5, The University of Arizona, Tucson, AZ, United States; 2 Graduate Interdisciplinary Program in Physiological Sciences, The University of Arizona, Tucson, AZ, United States; 3 Department of Pharmacology, College of Medicine, The University of Arizona, Tucson, AZ, United States; 4 Department of Neurology, College of Medicine, The University of Arizona, Tucson, AZ, United States; 5 Barrow Neurological Institute at Phoenix Children’s Hospital, The University of Arizona College of Medicine—Phoenix, Phoenix, AZ, United States; 6 Department of Child Health, The University of Arizona College of Medicine—Phoenix, Phoenix, AZ, United States; 7 Phoenix Veteran Affairs Health Care System, Phoenix, AZ, United States; 8 Department of Biological Sciences, University of Bath, Bath, United Kingdom; 9 College of Nursing, University of Arizona, Tucson, AZ, United States; 10 Department of Integrative Physiology, University of Colorado Boulder, Boulder, CO, United States

**Keywords:** PAC1, VPAC1/VPAC2 receptors, neurodegeneration, neuroinflammation, blood-brain barrier, 6-hydroxydopamine

## Abstract

There is an unmet clinical need for curative therapies to treat neurodegenerative disorders. Most mainstay treatments currently on the market only alleviate specific symptoms and do not reverse disease progression. The Pituitary adenylate cyclase-activating polypeptide (PACAP), an endogenous neuropeptide hormone, has been extensively studied as a potential regenerative therapeutic. PACAP is widely distributed in the central nervous system (CNS) and exerts its neuroprotective and neurotrophic effects *via* the related Class B GPCRs PAC1, VPAC1, and VPAC2, at which the hormone shows roughly equal activity. Vasoactive intestinal peptide (VIP) also activates these receptors, and this close analogue of PACAP has also shown to promote neuronal survival in various animal models of acute and progressive neurodegenerative diseases. However, PACAP’s poor pharmacokinetic profile (non-linear PK/PD), and more importantly its limited blood-brain barrier (BBB) permeability has hampered development of this peptide as a therapeutic. We have demonstrated that glycosylation of PACAP and related peptides promotes penetration of the BBB and improves PK properties while retaining efficacy and potency in the low nanomolar range at its target receptors. Furthermore, judicious structure-activity relationship (SAR) studies revealed key motifs that can be modulated to afford compounds with diverse selectivity profiles. Most importantly, we have demonstrated that select PACAP glycopeptide analogues (**2LS80Mel** and **2LS98Lac**) exert potent neuroprotective effects and anti-inflammatory activity in animal models of traumatic brain injury and in a mild-toxin lesion model of Parkinson’s disease, highlighting glycosylation as a viable strategy for converting endogenous peptides into robust and efficacious drug candidates.

## 1 INTRODUCTION

Traumatic brain injury (TBI) and Parkinson’s disease (PD) are particularly problematic neurological disorders due to a lack of curative therapies and the global burden on health care systems. It is projected that in the US alone some 1.2 million people will be affected by PD by 2030 (Marras et al., 2018). Parkinson’s disease is the second most common neurodegenerative disorder. None of the available therapies slow, prevent, or reverse the progression of PD (Smith et al., 2012), which inevitably leads to impaired cognition, severe motor skill deficits, heightened neuroinflammation, and dysregulation of brain homeostasis (Mattson, 2000). The cardinal PD motor deficits and some of the cognitive symptoms stem from dopaminergic (DA) neuronal cell death in the *substantia nigra pars compacta* (SNc), which is caused by extensive oxidative damage leading to mitochondrial dysfunction, endosomal and lysosomal deficits (Moore et al., 2005), as well as inflammatory response within the brain (Vázquez-Vélez and Zoghbi, 2021).

Traumatic brain injury (TBI) is a debilitating neurological disorder that affects roughly 69 million people worldwide each year (Dewan et al., 2019). Common clinical characteristics of TBI include cognitive and motor skill deficits, confusion, temporary loss of consciousness, and coma in severe cases (Andriessen et al., 2010). An external head injury can be followed by the activation of a cascade of secondary injury mechanisms including increases in pro-inflammatory cytokines, increased reactive oxygen species (ROS) and free radical production, excitotoxicity, mitochondrial dysfunction, and reduced concentrations of adenosine triphosphate (ATP), all of which further exacerbate and contribute to neurodegeneration and inflammation (Greve and Zink, 2009; Andriessen et al., 2010). TBI can result in a phenomenon known as post-traumatic sleep, wherein sleeping patterns/behavior are altered and often result in excess daytime fatigue and sleepiness (Rowe et al., 2014c). None of the available treatments have been clinically successful for TBI to date (Hall et al., 2010; Xiong et al., 2010; McConeghy et al., 2012; Tsang and Whitfield, 2012; Algattas and Huang, 2013). Therefore, novel restorative and anti-inflammatory treatments for TBI are necessary.

**FIGURE 1 |:**
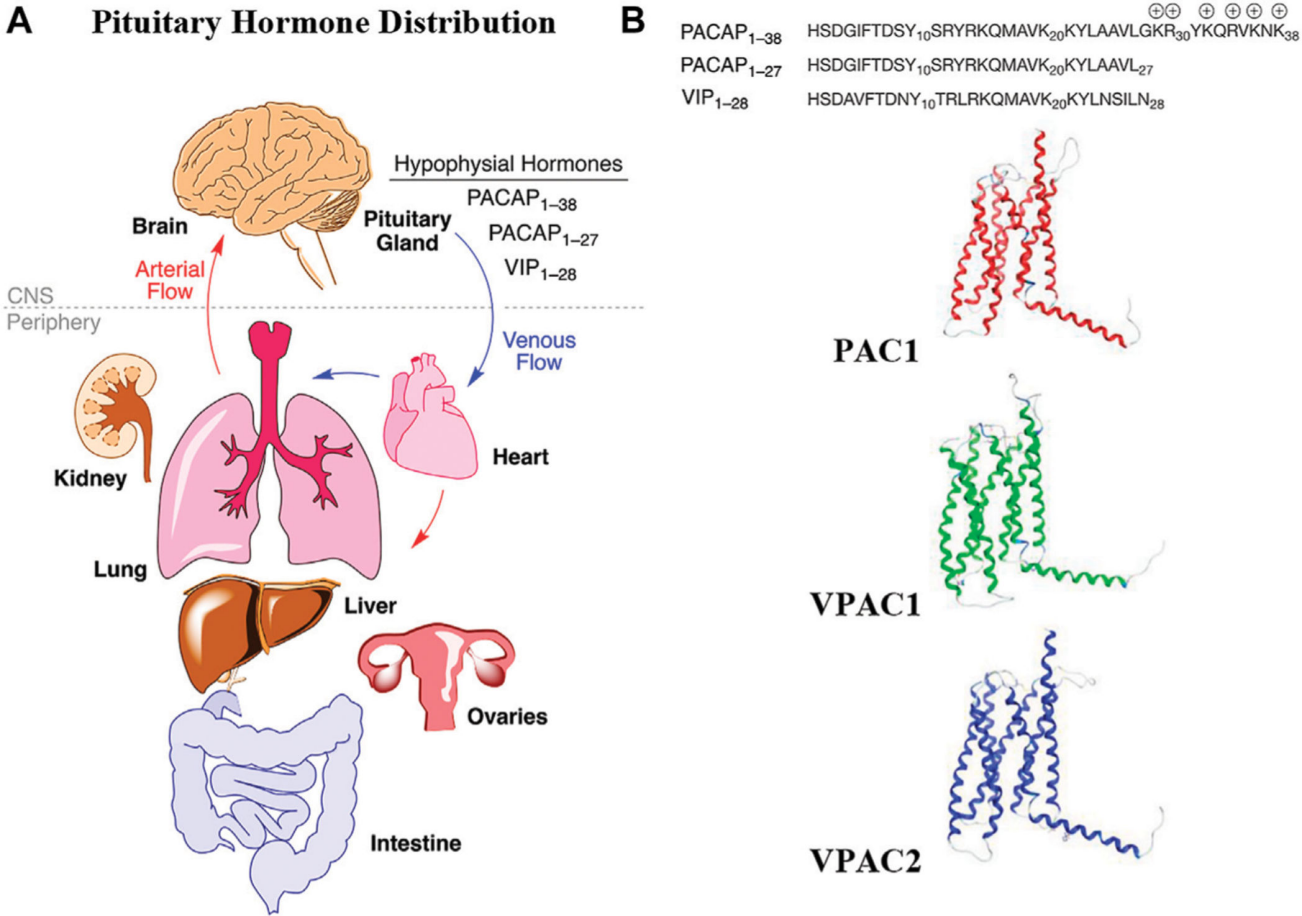
Hypophysial hormones distribution to peripheral organs and brain by the circulatory system. **(A)** Numerous peptides (hypophysial hormones) are secreted by the pituitary gland, which are recirculated throughout the body, and which have effects in all of the organs, including brain. Effects within the brain are dependent upon BBB penetration rates. **(B)** The relevant endogenous hormones, PACAP_1–38_, PACAP_1–27_, and VIP_1–28_ interact with 3 related Class B GPCRs, whose transmembrane structures are depicted as homology models in the inactive state.

Pleiotropic endocrine (hypophysial) hormones (Moody et al., 2011) secreted from the pituitary gland into the vascular system help influence growth, blood pressure, energy metabolism, glucose regulation and many other processes in organs throughout the body (Figure 1A). It should be noted that the brain must “*stand at the end of the line*” with all the other organs to receive peptide hormones secreted from the pituitary, a pea-sized gland located underneath the hypothalamus, and only after being pumped through the heart and filtered through the lungs. Of the 3 related hormones that form the basis for this study (Figure 1B), only PACAP_1–38_ with its highly charged C-terminal tail has any measurable brain penetrance. The presence of oxygen-sensitive methionine in the center of the helix in each of these hormones may be a “regulatory switch” that senses oxygenation states of the organism (Kim et al., 2014), and the lung, with its extensive surface area could serve as an oxidative “processing center” for the hypophysial hormones (Janssen-Heininger et al., 2008). Vasoactive intestinal peptide (VIP) and the PACAPs can influence energy management, sexual function, thyroid function and metabolism, as well as aspects of pregnancy, childbirth and breastfeeding, ion concentration at the kidneys, body temperature regulation and pain relief (Vaudry et al., 2009).

Numerous studies suggest that endogenous and/or exogenous PACAP promotes healing and cell survival (Dejda et al., 2005; Vaudry et al., 2005; Dejda et al., 2008; Botia et al., 2008; Manecka et al., 2013), especially within the CNS. Native PACAP has been demonstrated to be neuroprotective in diverse animal models of neurodegenerative disorders and neurological insults including Alzheimer’s disease (Onoue et al., 2002; Kojro, 2006; Rat et al., 2011), Parkinson’s disease (Reglodi et al., 2004a; Reglodi et al., 2004b), cerebral ischemia (Chen et al., 2006; Dejda et al., 2011), TBI (Farkas et al., 2004; Kóvesdi et al., 2008; Bukovics et al., 2014), and alcohol toxicity (Vaudry et al., 2002; Vaudry et al., 2005; Botia et al., 2011). PACAP stimulates neurite outgrowth in PC12 cells, demonstrating its neurotrophic potential (Deutsch and Sun, 1992; Tatsuno et al., 1992; Lazarovici et al., 1998; Manecka et al., 2013). Although PACAP is a promising therapy for neurodegenerative disorders, it suffers from poor stability *in vivo* and exhibits limited penetration of the blood-brain barrier (BBB) (Banks et al., 1993; Banks et al., 1998; Nonaka et al., 2005; Li et al., 2007; Rhea et al., 2018; Ghanizada et al., 2019).

Several strategies have been implemented to improve the stability of bioactive peptides including cyclization, *N*-methylation, lipidation, and PEGylation (Erak et al., 2018). As a result, a number of peptide-based drugs have entered the market with hundreds more in preclinical studies (Muttenthaler et al., 2021). Despite recent success in peptide drug development, the chemical strategies mentioned above are not general methods for enhancing membrane permeability, which is problematic for peptide drugs targeting the brain. Glycosylation addresses this challenge and has now been widely demonstrated to improve the stability, BBB permeability, and overall biological activity of peptide-based drugs (Witt et al., 2001; Egleton et al., 2005; Polt et al., 2005; Jones and Polt, 2015; Apostol et al., 2020). Carbohydrates can act as steric shields, effectively blocking proteases and peptidases from cleaving amide bonds proximal to the carbohydrate moiety, and glycosylation can also modulate the amphipathic character of peptides and influence how they interact with biological membranes (Lowery et al., 2007; Li et al., 2014; Lefever et al., 2015). Endogenous neurotransmitters are typically highly amphipathic in nature, and many of them interact strongly with the surface of cell membranes in an α-helical conformation (Epand et al., 1995; Shai, 1999). The carbohydrate residue increases water solubility and allows the peptide to dissociate reversibly from the membrane surface into the aqueous phase temporarily where it can adopt an ensemble of random-coil conformations.^1^ Thus, glycopeptides can “hop” along the cell membrane surface and efficiently sample various membrane compartments for target receptors (Lowery et al., 2007). With regards to penetration of the BBB, it has been hypothesized that glycosylation allows the glycopeptide to escape from the membrane following transcytosis (Poduslo and Curran, 1994; Banks et al., 1997; Fillebeen et al., 1999). Once a critical glycopeptide concentration is reached, the membrane will invaginate to form vesicles in which the glycopeptides can travel from one side of the BBB to the other, and remain topologically “outside” the endothelial cells. Thus, we envision that this glycosylation strategy will yield neuroprotective and BBB permeable PACAP analogues with improved *in vivo* stability. Earlier opioid glycopeptide drug candidates required continual receptor occupancy for pain relief, (Do Carmo et al., 2008; Lowery et al., 2011). Yet the effects of glycopeptides related to angiotensin_1–7_ are produced after only a few minutes of receptor occupancy, and can be permanent, as they affect cell development (Hay et al., 2019), and both PACAP and VIP are believed to be similar in this respect (Dejda et al., 2005).

PACAP was originally isolated from ovine hypothalamic tissues and was found to stimulate cAMP production in rat pituitary cells (Miyata et al., 1989). Sequence analysis revealed that the first isolated PACAP contained 38 amino acid residues and a highly charged amidated C-terminus. A shorter PACAP isoform containing only 27 amino acid residues was later discovered in side fractions during purification of PACAP_1–38_ (Miyata et al., 1990). Immunohistochemical studies revealed that PACAP is widely distributed in the central nervous system (CNS), and is also present in peripheral organs including the lung, heart, gut, and testis (Arimura et al., 1991; Masuo et al., 1993; Piggins et al., 1996; Vaudry et al., 2009), highlighting PACAP’s important physiological roles. The shorter PACAP_1–27_ exhibits 68% sequence homology with the VIP, making PACAP a member of the glucagon/secretin/growth hormone-releasing hormone superfamily of peptide ligands. These peptide ligands bind and activate Class B GPCRs, namely PAC1, VPAC1, and VPAC2. Class B GPCRs are structurally distinct from other GPCR families due to the presence of a large conserved extracellular N-terminal domain (Wu et al., 2017). The PAC1 receptor exhibits high affinity for PACAP but lower affinity for VIP, and the VPAC1 and VPAC2 receptors bind both ligands with high affinity (Laburthe et al., 2007; Dickson and Finlayson, 2009). The “mixed agonism” displayed by these hormones mimics the MOR/DOR/KOR receptor promiscuity displayed by the enkephalins, endorphins and dynorphins (Li et al., 2012). Several studies indicate that the neuroprotective and neurotrophic effects of PACAP are primarily mediated through the PAC1 receptor, while activation of the VPAC1 receptor may mediate inflammatory responses (Leceta et al., 2000). Activation of the VPAC2 receptor can lead to undesirable side effects including extensive vasodilation and intestinal water retention (Warren et al., 1992; Tsutsumi et al., 2002; Fizanne et al., 2004). Thus, adjustment of the native receptor selectivity may be an important consideration in developing ideal therapies based on this hormone system. It may also be important to activate more than one receptor simultaneously (Schiller, 2010).

Initial PACAP structure-activity relationship (SAR) investigations examined the importance of N- and C-terminal residues in receptor activation and binding, respectively. Because of the high degree of similarity in the N-terminal region amongst various Class-B GPCR ligands, it was postulated that sequential amino acid deletions at the N-terminus would drastically reduce biological activity. In line with this hypothesis, it was found that N-terminally truncated analogues of PACAP_1–38_ and PACAP_1–27_ were less potent agonists than the native peptides, eventually leading to the discovery of the PAC1 receptor antagonist PACAP_6–38_ (Robberecht et al., 1992a; Robberecht et al., 1992b). Interestingly, it was later found that deletion of the first 9 to 13 residues yielded analogues that re-gained agonist activity (Vandermeers et al., 1992). SAR experiments investigating the importance of the C-terminal residues of PACAP_1–38_ as compared to PACAP_1–27_ revealed that the C-terminal portion was responsible for initial receptor binding and recognition (Gourlet et al., 1996). Conformational analysis using NMR and circular dichroism in conjunction with strategic amino acid substitutions revealed that a C-terminal α-helix is important for receptor binding and recognition, whereas a β-turn-like conformation is required for receptor activation at the N-terminus (Inooka et al., 1992; Schafer et al., 1999; Inooka et al., 2001; Bourgault et al., 2008; Bourgault et al., 2009a; Bourgault et al., 2009b; Bourgault et al., 2011; Doan et al., 2011; Ramos-Álvarez et al., 2015). Additional structural information has been elucidated following the recent determination of cryo-EM structures of the PAC1 and VPAC1 receptors with PACAP bound, providing valuable information for future PACAP drug design (Duan et al., 2020; Kobayashi et al., 2020). Although previous PACAP-related drug discovery efforts have elucidated important factors that determine receptor activation and selectivity, none of these SAR studies investigated the effects of glycosylation for enhancing PACAP’s PK/PD and BBB transport properties *in vitro* and *in vivo*. Herein we describe our efforts towards the design, synthesis, and biological evaluation of a library of PACAP-derived glycopeptides.

**FIGURE 2 |:**
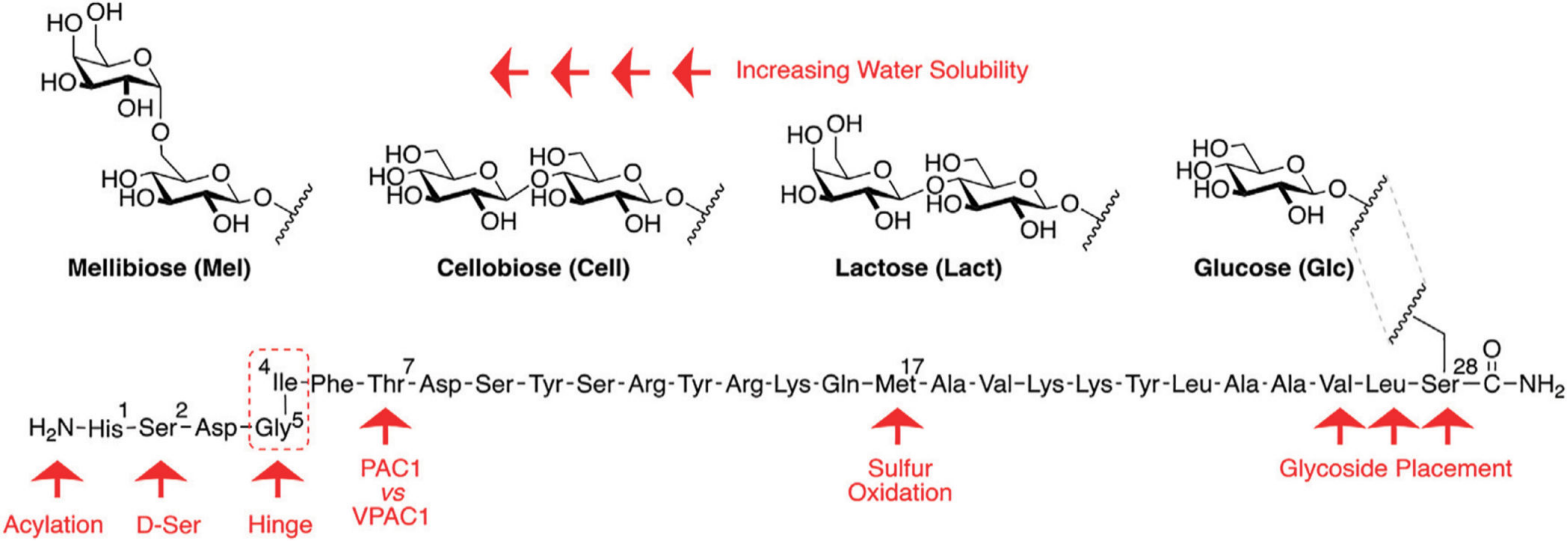
PACAP glycopeptide drug design. We hypothesized that the introduction of a carbohydrate moiety at the C-terminal end of PACAP1–27 could improve stability and BBB permeability. We envisioned that replacement of Met^17^ with an alkyl side chain-containing amino acid and stabilizing substitutions at positions 1 and 2 would also enhance stability. Furthermore, we postulated that strategic amino acid substitutions at positions 4, 5, and 7 could lead to improved receptor selectivity profiles.

**FIGURE 3 |:**
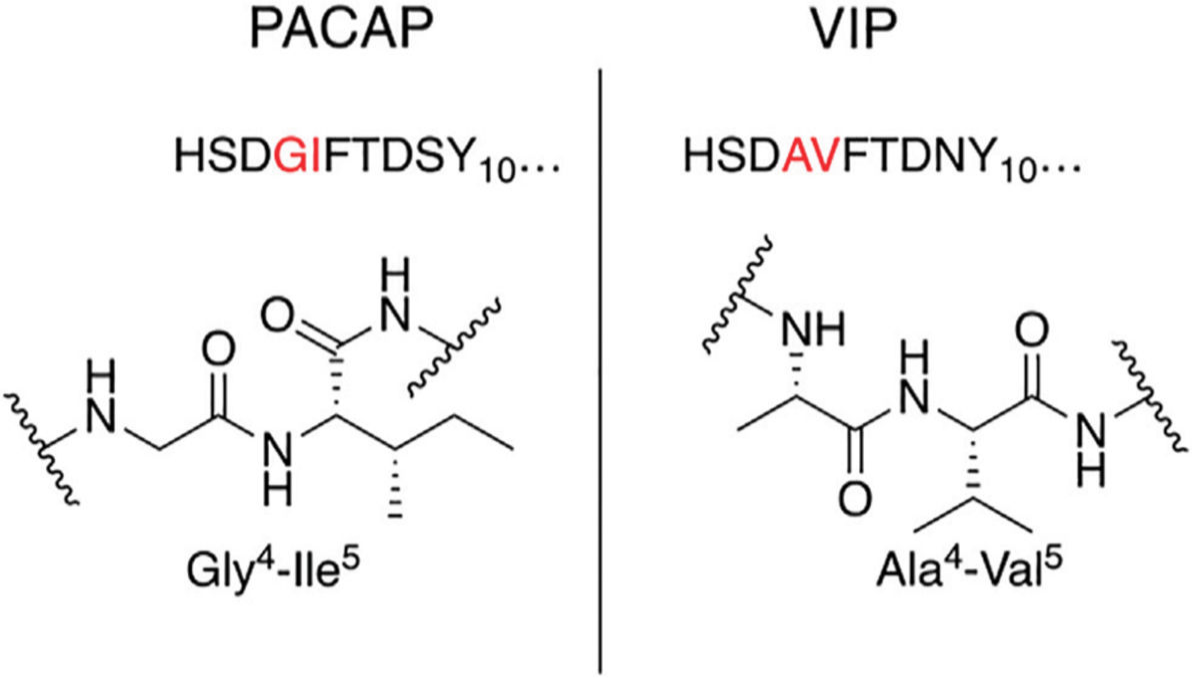
Hinge region comparison of PACAP and VIP. PACAP and VIP share a high primary amino acid sequence similarity but begin to diverge at positions 4 and 5. This relatively flexible “hinge” region allows the amphipathic helix to bind to the extracellular N-terminal domain of the Class B GPCR, and simultaneously interact with the transmembrane portion of the receptor. This region is sensitive to steric interactions between the 4^th^ and 5^th^ residues, and greatly influences selectivity of PAC1 and VPAC1 vs. VPAC2.

Our general design approach for the development of novel glycosylated PACAP analogues is highlighted in Figure 2. In addition to glycosylation, we introduced additional strategic amino acid substitutions to enhance stability and fine-tune receptor selectivity while preserving efficacy and potency. It is important to note that we chose PACAP_1–27_ as the template for our glycosylated analogues for several reasons. First, the extended C-terminal tail in PACAP_1–38_ facilitates anchorage of the peptide onto dipeptidyl peptidase IV (DPPIV), a membrane-bound protease that is known to cleave the N-termini of peptide hormones (Zhu et al., 2003). More importantly, it has been demonstrated that PACAP_1–38_ can induce migraine in healthy humans (Vollesen et al., 2018; Ghanizada et al., 2019; Haanes and Edvinsson, 2019). We focused our initial efforts primarily at the N- and C-terminal ends of PACAP_1–27_. We envisioned that the introduction of a carbohydrate moiety at the C-terminus of PACAP_1–27_ would improve BBB permeability and stability while only marginally affecting efficacy and potency. We also investigated the replacement of Met^17^, which is easily oxidized, with amino acids containing alkyl side chains including Leu and norvaline (Nva). We additionally examined the effect of substituting Thr^7^ with an Ala residue, which was shown to result in PAC1/VPAC1 selective agonists (Doan et al., 2011). We then focused our attention to the N-terminal region of PACAP, which is responsible for receptor activation. Interestingly, His^1^-Asp^3^ are common amino acid residues between PACAP and VIP, but their primary sequences begin to diverge at positions 4 and 5. In PACAP, positions 4 and 5 are occupied by the dipeptide motif Gly^4^-Ile^5^, while the analogous positions in VIP are occupied by Ala^4^-Val^5^ (Figure 3). The Gly^4^-Ile^5^ motif in PACAP is more conducive for β-turn conformations, whereas the Ala^4^-Val^5^ moiety has a high propensity for α-helix formation. Furthermore, Val and Ile contain branched alkyl side chains with slightly different steric profiles. Thus, we hypothesized that this “hinge region” may be the key to discriminate between the PAC1 and VPAC1/VPAC2 receptors. This prompted us to explore the introduction of α-helix or β-turn inducing motifs and amino acids with alkyl side chains containing differing steric profiles in this region and the resultant effects on receptor selectivity. We also set out to introduce substitutions and modifications at His^1^ and Ser^2^ due to their involvement in the *in vivo* stability of PACAP and other related peptide hormones. Thus, we introduced stabilizing substitutions in this region including replacement of Ser^2^ with D-Ser, L-Thr, or D-Thr, and the introduction of an N-acyl group at the α-amino nitrogen of His^1^.

**FIGURE 4 |:**
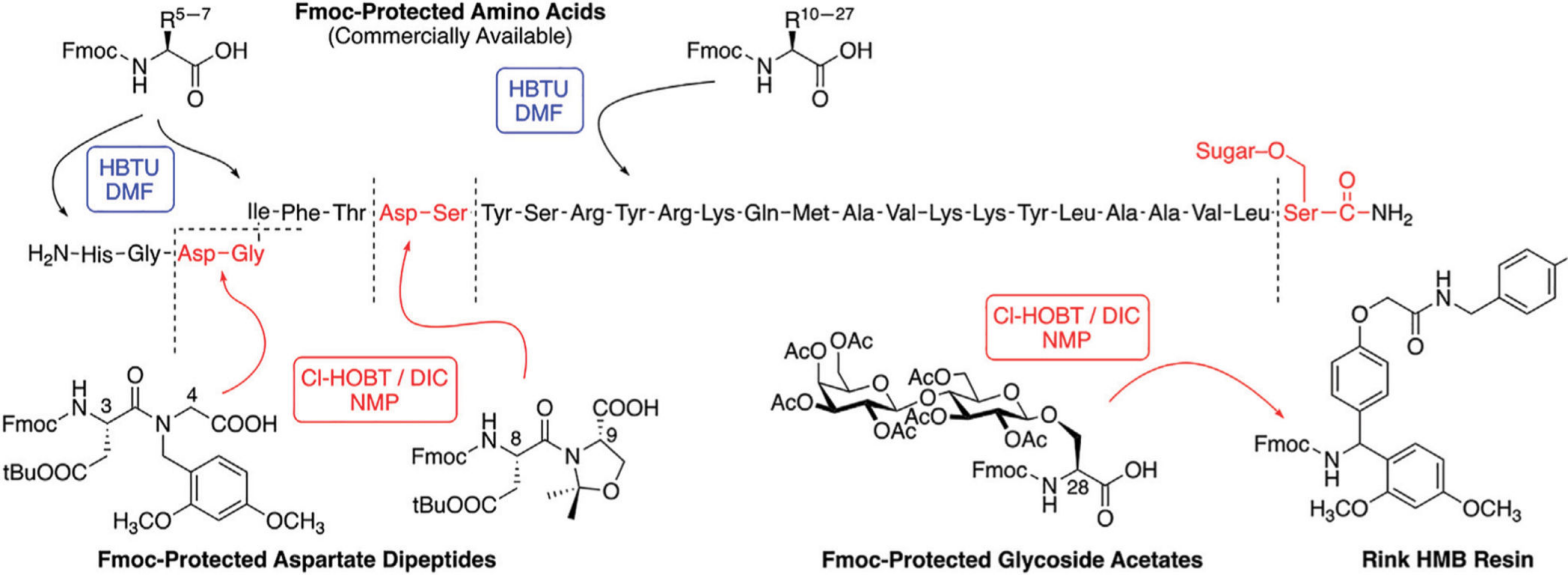
Synthetic scheme for preparation of PACAP-Derived glycopeptide.

We have synthesized a modest library of PACAP glycopeptides that were purified by reversed phase high performance liquid chromatography (RP-HPLC) and characterized by electrospray ionization mass spectrometry (ESI-MS). Our synthetic approach for the preparation of these glycopeptides detailed in Figure 4. The structures of the PACAP-derived glycopeptides are summarized in Table 1 and their associated HPLC data and MS data are listed in Table 2. All of the compounds were evaluated *in vitro* for their ability to stimulate cAMP production in CHO-cells expressing the PAC1, VPAC1, or VPAC2 receptors. Selected analogues were evaluated for BBB transport and *in vivo* stability using *in vivo* microdialysis coupled to mass spectrometry. Finally, the analogues **2LS80Mel** and **2LS98Lac** were evaluated for their neuroprotective potential and anti-inflammatory activity in a rodent model of TBI and the progressive 6-OHDA hemi-parkinsonian rat model, respectively.

## 2 MATERIALS AND METHODS

### 2.1 Peptide Synthesis and Purification

#### 2.1.1 General

Peptide synthesis was performed on a Prelude® automated peptide synthesizer. Synthesis was performed either in an automated fashion or semi-manually where reagents were loaded into the reaction vessels using a syringe. The resin was agitated (mixed) using a steady flow of argon. The washing steps with DMF and DCM were performed for 2 min each.

#### 2.1.2 Rink Amide Resin Preparation

0.5 mmol of Rink Amide-MBHA resin (1g, ds: 0.5 mmol/g) was placed in a 45 ml reaction vessel and swelled in dimethylformamide (DMF) for 1 h. Fmoc removal was achieved by addition of a solution containing 2% DBU-3% piperidine in DMF (10 ml) and mixing for 4 min. The mixture was then drained, and the resin was washed once with 10 ml of DMF. Fmoc removal was then repeated for an additional 8 min followed by 6 DMF washes (10 ml, 2 min).

#### 2.1.3 Glycosyl Amino Acid Loading

0.6 mmol (1.3 eq.) [N-(9-fluorenylmetoxycarbonyl)-L-serine-3-yl] peracetyl-β-O-glucopyranoside and 0.65 mmol (1.3 eq.) 6-Cl-HOBt were placed into a vial and dissolved in 8 ml N-methylpyrrolidinone (NMP). 0.65 mmol (1.3 eq.) of N,N′-diisopropylcarbodiimide (DIC) was then added into the solution. The mixture was vortexed for 1 min and then added to the resin. The reaction mixture was mixed overnight for 16 h. The mixture was diluted with DMF (10 ml) and drained immediately. Then the resin was washed 6 times with DMF (10 ml) and then 4 times with dichloromethane (DCM). The unreacted NH_2_ sites on the resin were then capped with a solution of 10% N,N-diisopropylethylamine and 10% Ac_2_O in 8 ml DCM. This reaction was allowed to proceed for 1 h. The resin was then washed 6 times with DCM and then washed 4 times with DMF to prepare the resin for the next automated steps.

#### 2.1.4 Prelude® Automated Synthesis

The Leu^27^-Tyr^10^ amino acid series was prepared using the automated SPPS feature on the Prelude® automated peptide synthesizer. The Fmoc group was removed as described above and a solution containing the desired Fmoc-amino acid (3 equivalents), N,N,N′,N′-tetramethyl-O-(1H-benzotriazol-1-yl) uronium hexafluorophosphate (HBTU) (3 equivalents), and *N*-methylmorpholine (12 equivalents) was loaded to the resin. The reaction mixture was mixed for 30 min followed by a single DMF wash (10 ml). The coupling reaction was repeated a second time for 30 min, and the resin was then washed 6 times with DMF. Subsequent deprotection and coupling cycles were then performed up to Tyrosine^10^.

#### 2.1.5 Manual Loading of DS Dipeptide

The Fmoc group was initially removed as described above. Then, 1 mmol of Fmoc-DS-OH or Fmoc-DG-OH (2 equiv.) and 1 mmol of 6-Cl-HOBt (2 equiv.) were added to a vial and dissolved in 8 ml of NMP. 1 mmol of DIC (2 equiv.) was then added to the solution. The mixture was vortexed for 1 min and then added to the resin. The reaction mixture was mixed for 40 min. The resin was then washed once with DMF (10 ml) and a second coupling was performed for 60 min. The mixture was diluted with DMF (10 ml) and drained immediately. Then the resin was washed 6 times with DMF (10 ml).

#### 2.1.6 Automated Addition of IFT

The Ile^5^-Thr^7^ amino acid series was prepared using the automated SPPS feature on the Prelude® automated peptide synthesizer. The Fmoc group was removed as described above and a solution containing the desired Fmoc-amino acid (3 equivalents), HATU (3 equivalents), and 2,4,6-trimethylpyridine (12 equivalents) in 10 ml of DMF was loaded to the resin. The reaction mixture was mixed for 30 min followed by a single DMF wash (10 ml). The coupling reaction was repeated a second time for 30 min, and the resin was then washed 6 times with DMF. Subsequent deprotection and coupling cycles were then performed up to Isoleucine^5^.

#### 2.1.7 Manual Loading of Amino Acids

The Fmoc group was initially removed as described above. Then, 1.5 mmol amino acid (3 equiv.) and 1.5 mmol of 6-Cl-HOBt (3 equiv.) were added to a vial and dissolved in 8 ml of NMP. 1.5 mmol of DIC (3 equiv.) was then added to the solution. The mixture was vortexed for 1 min and then added to the resin. The reaction mixture was mixed for 40 min. The resin was then washed once with DMF (10 ml) and a second coupling was performed for 60 min. The mixture was diluted with DMF (10 ml) and drained immediately. Then the resin was washed 6 times with DMF (10 ml). After the final amino acid the Fmoc group was initially removed as described above.

#### 2.1.8 Acetyl Cleavage

120 ml of a 50% solution containing NH_2_NH_2_ × H_2_O in NMP (10 ml per reaction vessel) was prepared and added to the resin. The solution was mixed overnight for 16 h. The solution was then drained, and a second 10 ml portion of 50% NH_2_NH_2_ × H_2_O was added to each reaction vessel. This solution was mixed for an additional 2 h. The 50% NH_2_NH_2_ × H_2_O was then drained and the resin was washed 8 times with DMF (10 ml), 8 times with DCM (10 ml), and dried under vacuum for 3 h.

#### 2.1.9 Cleavage From the Resin and Global Side Chain Deprotection

The dried resin was treated with an acidic cleavage cocktail containing anhydrous trifluoroacetic acid (TFA), DCM, H_2_O, triethylsilane, and anisole (90:10:2:3:0.5). The resin was mixed for 1 h, and the solution was collected into a 45 ml centrifuge tube. The cleavage step was repeated 2 more times for 10 min periods. The combined fractions were slowly evaporated over a stream of argon until the peptide began to crash out. Cold ether (∼40 ml) was then added to precipitate the peptide and the mixture was centrifuged for 10 min at 5 G. The ether layer was decanted off and ether (∼40 ml) was added to the crude peptide and centrifuged once more. This process was repeated for a third time. After decanting the ether layer, the crude peptide was dried under vacuum overnight.

#### 2.1.10 HPLC Purification and Characterization of Crude Peptides

These crude samples were then purified on a Gilson system with a UV detector (at 280 nm) using a Vydak C18 preparative reversed-phase column (250 mm × 50 mm) using a gradient of 5–80% CH_3_CN vs. 0.1% CF_3_COOH in H_2_O over 60 min to give the glycopeptides in pure form, assessed for purity by analytical HPLC (Inspire C18 5 μm 250 mm × 4.6 mm column) on a Varian LC with a diode array detector system (at 280 nm) employing the same gradient over a period of 15 min. The pure fractions obtained from preparative HPLC purification were frozen at −80°C and then lyophilized to afford the pure peptides as white and fluffy solids. The pure peptides were then characterized using mass spectrometry (ESI-MS).

#### 2.1.11 *In Vitro* cAMP Stimulation in CHO Cells Expressing PAC1, VPAC1, and VPAC2 Receptors

The cell lines, culture, and cAMP assay are all described in our previous work (Apostol et al., 2021). Briefly, human PAC1, VPAC1, and VPAC2 receptors expressed in CHO cells were used for all experiments. Cells were grown in 50:50 DMEM-F12 medium with 10% heat-inactivated fetal bovine serum, 1X penicillin-streptomycin supplement, and 500 μg/ml G418 (neomycin) to maintain selection (all from ThermoFisher) at 37°C with 5% CO_2_ atmosphere. For the cAMP assay, cells were seeded at 20,000 cells/well in a 96 well plate, recovered overnight, then serum starved for 4 h. The cells were then incubated with 500 μM IBMX for 20 min, followed by agonist concentration curves in 500 μM IBMX media for 10 min. The reaction was terminated, boiled, then collected, followed by competition with 7 μg of bovine protein kinase A (Sigma-Aldrich) and ∼1 pmol of ^3^H-cAMP (PerkinElmer). The assay was incubated at room temp for 1 h, then collected onto GF/B filter plates and the data read using a MicroBeta2 scintillation counter (PerkinElmer). The resulting data was fit to a 3 variable non-linear regression curve using GraphPad Prism, which resulted in potency (EC_50_) and efficacy (E_MAX_) measurements. Native PACAP_1–27_ was used as a positive control, and also used to define an E_MAX_ level of 100%.

#### 2.1.12 *In Vitro* Stability in Rat Serum and aCSF of PACAP Glycopeptides

Compounds were added in rat serum or aCSF at concentration of ∼1 μM. Initial sample was obtained immediately after mixing the solution. Aliquots were taken at different time points then added in quenching solution that contains 10% acetic acid and internal standard. Quenched samples then were ZipTip* cleaned before being quantified by infusion mode of mass spectrometry, Thermo LTQ Orbitrap Velos. All concentrations at different time points were normalized to initial sample concentration which is considered 100% at time of 0 min.

#### 2.1.13 *In Vivo* Stability and BBB Transport Determination by Microdialysis Coupled to Mass Spectrometry

*In vivo* stability was measured by taking blood sample before and after i.v. injection at 1, 5, 10 and every 10 min, where time 0 min corresponds to injection time. Serum samples were obtained by spinning down blood samples for 5 min, then diluted 100 times for analysis. BBB transport was measure by collecting dialysate from microdialysis at flow rate of 0.5 μl/min. Dialysate samples were collected during every 10 min before and after injection. Both serum and dialysate samples were stored on dry ice or −80°C. Samples were ZipTip® cleaned before being quantified by nano liquid chromatography-tandem mass spectrometry. Self-packed 10∼15 cm analytical column (ID 75 μm, 3 μm particle, Reprosil-Pur 120 C18-AQ) and Thermo LTQ Orbitrap Velos were used for analysis.

### 2.2 *In Vivo* Traumatic Brain Injury Studies in Mice

#### 2.2.1 Rigor

Animal studies were conducted in accordance with the guidelines established by the National Institutes of Health (NIH) and the Institutional Animal Care and Use Committee (IACUC) at the University of Arizona. The Animal Research: Reporting *In Vivo* Experiments (ARRIVE) guidelines were followed (Kilkenny et al., 2010). Adult (∼3-month-old) male and female C57BL/6 mice (Harlan Laboratories, Inc., Indianapolis, IN) were used for all TBI experiments (*n =* 56). Mice were housed in a 14:10 light-dark period (lights on at 6:00AM; 200 lux cool, white fluorescent light) at a constant temperature (23°C ± 2) and given food and water *ad libitum*. All mice used in this study were singly housed as necessitated by the sleep cages. Following shipment, mice were acclimated to their environment for at least 1 week prior to any experiments. After surgery, mice were evaluated daily during post-operative care via physical examination and documentation of each animal’s condition. Animal care was approved by the Institutional Animal Care and Use Committee at the University of Arizona. Randomization of animals was achieved by assigning mice to treatment groups prior to initiation of the study to ensure equal distribution among groups. A power analysis was performed to identify group sizes that enable statistically robust detection of brain injury-induced deficits while minimizing the number of animals needed; this was based on preliminary data and previously published work from our group (Rowe et al., 2014c; Rowe et al., 2019). Data collection was stopped at pre-determined final endpoints based on days post-injury (DPI) for each animal. All animal behavior was scored by investigators blinded to treatment groups. All histology, flow cytometry, and cytokine analyses were performed by investigators blinded to the treatment groups. All behavior testing was conducted in the same room to avoid potential confounding of novel locations on behavioral responses.

#### 2.2.2 2LS80Mel Administration

30 min prior to TBI or sham injury, mice were randomly assigned to a therapeutic group and received an intraperitoneal injection of 2LS80Mel (10 mg/kg) or sterile saline (0.25 ml). Groups were as follows: TBI-2LS80Mel *n* = 17; TBI-vehicle *n* = 17; sham *n* = 22 (vehicle *n* = 11, 2LS80Mel *n* = 11).

#### 2.2.3 Midline Fluid Percussion Injury

Mice (19–24 g) were subjected to midline fluid percussion injury (mFPI) consistent with methods previously described (Lifshitz, 2009; Harrison et al., 2014; Rowe et al., 2014a; Rowe et al., 2014b; Rowe et al., 2014c). Mice were anesthetized using 5% isoflurane in 100% oxygen for 5 min and the head of the mouse was placed in a stereotaxic frame with continuously delivered isoflurane at 2.5% via nosecone. While anesthetized, body temperature was maintained using a deltaphase® isothermal heating pad (Braintree Scientific Inc., Braintree, MA). A midline incision was made exposing bregma and lambda, and fascia was removed from the surface of the skull. A trephine (3 mm outer diameter) was used for the craniectomy, centered on the sagittal suture between bregma and lambda without disruption of the dura. An injury cap prepared from the female portion of a Luer-Loc needle hub was fixed over the craniectomy using cyanoacrylate gel and methyl-methacrylate (Hygenic Corp., Akron, OH). The injury hub was closed using a Luer-Loc cap and mice were placed in a heated recovery cage and monitored until they were ambulatory before being returned to their respective sleep cages.

For injury induction 24 h post-surgery, mice were re-anesthetized with 5% isoflurane delivered for 3 min. The cap was removed from the injury-hub assembly and the dura was visually inspected through the hub to ensure it was intact with no debris. The hub was then filled with normal saline and attached to an extension tube connected to the male end of the fluid percussion device (Custom Design and Fabrication, Virginia Commonwealth University, Richmond, VA). An injury of moderate severity for our injury model (1.4 atm) was administered by releasing the pendulum onto the fluid-filled cylinder following the return of a toe-pinch response. Sham mice underwent the same procedure except the pendulum was not released. Injured mice were monitored for presence of a forearm fencing response and righting reflex times were recorded as indicators of injury severity (Hosseini and Lifshitz, 2009). The righting reflex time is the total time from the initial impact until the mouse spontaneously rights itself from a supine position. The fencing response is a tonic posturing characterized by extension and flexion of opposite arms that has been validated as an overt indicator of injury severity (Hosseini and Lifshitz, 2009). The injury hub was removed, and the brain was inspected for uniform herniation and integrity of the dura. The incision was cleaned with saline and closed using sutures. Moderately brain-injured mice had righting reflex recovery times greater than 4 min and a positive fencing response. Righting reflex times for TBI-2LS80Mel mice were 477.13 ± 83.13 s and the righting reflex times for TBI-vehicle mice were 417.05 ± 107.47 s. Mice subjected to a moderate mFPI regained gross neurological function with no intervention and therefore, these injuries were most consistent with mild-moderate TBI, with a Glasgow coma score (GCS) of 9–13, in which patients are generally responsive, but likely disoriented (Lifshitz et al., 2016). Sham mice recovered a righting reflex immediately when removed from the injury device (within 20 s). After spontaneously righting, mice were placed in a heated recovery cage and monitored until they were ambulatory (approximately 5–15 min) before being returned to their piezoelectric sleep cage (see below). Adequate measures were taken to minimize pain or discomfort.

#### 2.2.4 Collection of Sleep-Wake Parameters

Sleep and wake activity were measured using a non-invasive piezoelectric sleep cage system (Signal Solutions, Lexington, KY, United States), which classified sleep behavior according to previously described methods (Harrison et al., 2015; Rowe et al., 2018; Saber et al., 2020). This non-invasive method has been validated with electroencephalogram (EEG) and human observations, and has demonstrated a classification accuracy (sleep vs wake) of >90% in mouse sleep research (Mang et al., 2014). Each cage had an open bottom that allowed direct placement on a Polyvinylidine Difluoride sensor on the cage floor, and the sensors were coupled to an input differential amplifier to generate pressure signals. Sleep was characterized by regular breathing movements associated with sleep (3 Hz, regular amplitude signals) (Donohue et al., 2008). Mice that were characterized as exhibiting wake behavior had the absence of this sleep signal and the presence of higher amplitude, irregular spiking, associated with volitional movements. The piezoelectric signals were analyzed over tapered 8 s windows at a 2 s interval, from which a decision statistic was computed and classified by a linear discriminate classifier as “sleep” or “wake”. Sleep bouts required a minimum of 4 s epochs. Data were also collected for cumulative minutes slept within each 24 h period and were evaluated separately in the dark period when mice are typical awake. To assess sleep fragmentation, the frequencies of individual sleep bouts with different episode durations were analyzed (Rowe et al., 2018; Giordano et al., 2020). Sleep bouts were assigned to one of eight bins of exponentially increasing durations (4–7, 8–15, 16–31, 32–63, 64–127, 128–255, 256–511, and >512 s) and the frequency of the number of bouts in each bin was calculated. Sleep from 7 mice had a poor piezoelectric signal and were excluded from analyses. Groups sizes for sleep: TBI-2LS80Mel *n* = 14; TBI-vehicle *n* = 17; sham *n* = 18.

#### 2.2.5 Rotarod

Sensorimotor function was assessed using the Rotor-Rod system (San Diego Instruments) as we have previously published (Rowe et al., 2018). Mice were acclimated 3 days prior to surgery/injury. The mice were placed on the stationary rod and allowed to explore for 30 s. Following exploration, the mice were placed on the rod at a constant speed of 5 revolutions per minute (rpm). If the mouse fell off the rod, it was placed back on the rod and the timer was restarted (until the mice could walk 15 s at 5 rpm). Next, mice were placed on the rod with an initial rotation speed of 5 rpm and an acceleration of 0.2 rpm/sec. The trial ended when the mouse fell off the rod; the acclimation period ended after two trials. Following acclimation, mice were trained over three consecutive days prior to surgery/injury and the last training session was recorded as baseline. Testing occurred at 2, 5, and 7 DPI. For the training and testing phase, mice were placed on the stationary rod and the motor was started at 5 rpm with an acceleration of 0.2 rpm/sec. Two trials were run back-to-back and mice were returned to cages thereafter. After 10 min, mice preformed a third trial. Time spent on the rotarod from the best two trials were averaged to generate a time score for each mouse.

#### 2.2.6 Neurological Severity Score

Neurological impairments were assessed at 2, 5, and 7 DPI using an 8-point NSS paradigm adapted from those previously used in experimental models of TBI and previously reported by our lab (Chen et al., 1996; Semple et al., 2010; Pleasant et al., 2011; Ziebell et al., 2011; Rowe et al., 2018; Rowe et al., 2019). One point was given for failure on an individual task, whereas no points were given if a mouse completed a task successfully. Mice were observed for hind limb flexion, startle reflex, and seeking behavior (presence of these behaviors was considered successful task completion). Mice traversed in sequence, three, two, and one-centimeter beams. The beams were elevated, and mice were given 1 min to travel 30 cm along the beams. The task was scored as a success if the mouse traveled 30 cm with normal forelimb and hindlimb position (forelimb/hindlimb did not hang from the beam). Mice were also required to balance on a 0.5 cm beam and a 0.5 cm round rod for 3 s in a stationary position with front paws between hind paws. The resulting non-parametric data are presented as a composite score ranging from zero to eight, representing performance on all tasks combined. High final NSS scores indicate task failure and are interpreted as neurological impairment.

#### 2.2.7 Novel Object Recognition

Cognitive impairment was tested at 14 DPI using the novel object recognition (NOR) test as previously published (Rowe et al., 2014b; Harrison et al., 2015; Saber et al., 2021). Mice were acclimated to clean individual plastic testing cages (30.5 cm W × 40.7 cm L × 20.3 cm H; Volume 0.025 m^3^) for 1 h. The test consisted of three phases: habituation, training, and testing. The same plastic cage was used for all three phases to prevent potential confounding of placing mice in a new cage for novel object testing. Two identical objects (plastic toys) were placed in opposing quadrants of the testing cage for the training phase. Mice were then placed in the center of the open field and given 5 min to explore the objects. Following training, mice were returned to their home cages. Testing began 4 h after training. One familiar object was placed in an original location and one novel object was placed in the opposing quadrant of the open field. Mice were placed into the center and given 5 min to explore. For testing, the times spent actively investigating the novel and familiar object were quantified. Investigation of an object included the mice sniffing, touching, or climbing onto an object while facing the object. If an animal climbed onto an object and sniffed into the air, this time was not calculated into the exploration of the novel object. Testing data are displayed as the percentage of total investigation time spent with each object and as a discrimination index (DI) in which $\;\text{DI}\;=\frac{\;\text{Tnovel}\;-\;\text{Tf}\;\text{amiliar}\;}{\;\text{Tnovel}\;+{\;\text{Tf}\;\text{amiliar}\;}^{*}}$

#### 2.2.8 Flow Cytometry Analysis of Blood

Whole blood (100 μl) was incubated with Fc block (1:500, BioLegend, San Diego, CA) for 10 min and then incubated with the following antibodies for 15 min: CD45 (1:200, BioLegend, San Diego, CA), CD11b (1:200, BioLegend, San Diego, CA), Ly6C (1:100, BioLegend, San Diego, CA), Ly6G (1:200, BioLegend, San Diego, CA), CD115 (1:100, BioLegend, San Diego, CA), NK1.1 (1:100, BD BioSciences, San Jose, CA), B220 (1:200, BioLegend, San Diego, CA), and Siglec F (1:100, BD BioSciences, San Jose, CA). Samples were then lysed with 1X Red Blood Cell Lysis Buffer (BioLegend, San Diego, CA) and washed with FACS buffer. Cell populations were sorted with a BD FACSCanto^™^ II Cell Analyzer (BD Biosciences, San Jose, CA). Cells were gated on CD45^+^ where a total of 80,000 events 80,000 events were collected. Cells were then gated on CD11b + events. To specifically focus on monocyte populations, cells were gated on Ly6C and CD115, and CD3, B220, Siglec F, and NK1.1 were used to gate out T cells, B cells, eosinophils, and natural killer cells, respectively. Populations of interest were defined as CD11b^+^Ly6C^high^ monocytes and CD11b^+^Ly6G^+^ neutrophils. The total number of events of interest were taken as a ratio to CD11b+ cells to calculate percentage of cells of interest. Live and Dead cells were gated out using 7-AAD. There were handling errors with four blood samples and those mice were removed from analyses.

#### 2.2.9 Cytokine Measurement

Approximately 75 μl of blood were centrifuged to collect approximately 40 μl of plasma from each animal at 2 and 14 days post-injury (Saber et al., 2020). Multiplex cytokine assays (MILLIPLEX MAP Mouse Cytokine/Chemokine Magnetic Bead Panel, Millipore Sigma, Burlington, MA) quantified interleukin (IL)-1β, IL-6, and tumor necrosis factor (TNF)-α in peripheral blood. Manufacturer’s instructions were followed. Briefly, supplied standards and plasma samples were incubated with antibodies coupled to magnetic beads for each analyte overnight at 4°C with agitation. Next, standards and samples were incubated with Detection Antibodies for 1 h at room temperature with agitation and then Streptavidin-Phycoerythrin for 30 min at room temperature with agitation. Fluorescent intensity was measured on a Bio-Plex® 200 system (Bio-Rad Laboratories, Hercules, CA) and corresponded to the concentration of each analyte in plasma samples. Duplicate values were averaged and analyzed between groups. Grubb’s outlier test identified four statistical outliers from cytokine data that were removed.

#### 2.2.10 Tissue Preparation

At 15 DPI we performed transcardial perfusions with phosphate buffered saline (PBS) and 4% paraformaldehyde, brains were harvested, and the hemispheres were separated. Brains were incubated in successive concentrations of sucrose (15%, 30%). Using the Megabrain technique (Green et al., 2018), 1 hemisphere per animal was frozen in groups of 5–9 in optimal cutting temperature compound (OCT) and cryosectioned, in the coronal plane at 40 μm thickness, and mounted on slides before staining.

#### 2.2.11 Iba1 Immunohistochemistry

Slides were baked for 3 h at 56°C prior to use. Brains were rehydrated in PBS and antigen retrieval was performed in sodium citrate buffer (pH 6.0). Slides were washed and blocking solution was applied (4% Normal horse serum [NHS], 0.1% Triton-100 in PBS). Following blocking, primary antibody solution (rabbit anti-Iba1; WAKO cat #019919741 at 1: 1,000 concentration in 1% NHS, 0.1% triton-100 in PBS) was allowed to bind overnight at 4°C. Slides were washed and secondary antibody solution (biotinylated horse anti-rabbit IgG (H + L); vector BA-1100 at 1:250 concentration in 4% NHS and 0.4% triton-100 in PBS) was applied and allowed to bind for 60 min. Endogenous peroxidases were blocked with H_2_O_2_ and ABC solution (Vectastain ABC kit PK-6100). Then, after washing, DAB solution (from Vector DAB peroxidase substrate kit SK-4100) was applied for 10 min. Tissue was dehydrated and cleared in ethanol and Citrisolv^™^ (Decon Labs, Inc.), respectively. Coverslips were applied using DPX mounting medium.

#### 2.2.12 Imaging and Analysis

A subset of mice was processed for immunohistochemistry: TBI-2LS80Mel *n =* 5; TBI-vehicle *n =* 7; sham *n* = 5. All immunohistochemistry was performed on 2–3 brain slices from each animal, and 3 images per slice were analyzed to reduce variability (6–9 images per animal). Images were taken in three regions of the cortex; the peri-injury site, primary somatosensory barrel field (S1BF), and the perirhinal cortex. Z-stack images of stained tissue were taken at 400x (×40 objective lens, ×10 ocular lens) using Zeiss Imager A2 microscope with AxioCam MRc5 digital camera with Neurolucida 360 software, with consistent brightness, numerical aperture and Z-stack height. Iba1 staining was analyzed using the skeletal analysis plugin following the protocol previously published (Morrison et al., 2017; Young and Morrison, 2018). Cell somas were counted manually. Total microglial count, process length and endpoints were recorded and averaged per number of cells in each region of interest.

### 2.3 *In Vivo* Hemi-Parkinsonian Rat Model

#### 2.3.1 Animal Care and Treatment

A total of 32 8-weeks old male Sprague Dawley rats (225 g at arrival) were purchased from Envigo RMC Inc. (Indianapolis IN). Rats were housed in temperature and humidity-controlled rooms, and allowed access to food and water *ad libitum*. Rats were kept on a 12 light/dark cycle, and all behavioral experiments were performed during the day portion of their light cycle. All animal studies were approved by the IACUC at The University of Arizona, were performed in accordance with the NIH Guidelines for the Care and Use of Laboratory Animals, and the ARRIVE guidelines.

#### 2.3.2 Experimental Design

Rats were treated with **2LS98Lac** or saline (vehicle) as outlined in the Schemes in Figures 9A,B via intraperitoneal (i.p.) injection (15 mg/kg). All animals were made hemi-parkinsonian with unilateral, striatal 6-hydroxydopamine (Sigma Aldrich, St. Louis, MO) injections. After surgery, behavioral assessments were done at 2- and 4-weeks post-surgery. 24 h after the last test animals were either euthanized and whole brains were harvested for future biochemical analysis or perfused and brains collected for immunohistochemistry and stereological assessment of tyrosine hydroxylase-positive (TH+) cells remaining in the SNc. In study 1: *n =* 16 each group were included, and all underwent the behavioral analysis: *n =* 16, and for the stereology in the SNc, *n =* 8 each group was used. In study 2: *n =* 23 each group were included. In study 2, 1 animal in the vehicle group was removed from all analyses, as the 6-OHDA injection point was found to be outside of the striatum, reducing the behavior (*n =* 22) and the stereology (*n =* 14) in this group. In the **2LS98Lac** group, one video at 4 weeks was lost, reducing *n =* 22 in this specific analysis, and for the stereology, 2 brains were damaged during processing and could not be included in the stereology of the SNc, therefore *n* = 13. For biochemical subanalyses, *n* = 8 each group. For the microglial morphology subanalysis *n* = 7 each group.

#### 2.3.3 Unilateral 6-Hydroxydopamine Stereotaxic Surgery

Rats were made hemi-parkinsonian through established protocols (Yue et al., 2014). Briefly, rats were anesthetized with isoflurane (1.5–2.0%; VetOne, Boise, ID) and fitted with a nose cone for continuous isoflurane administration. 6-OHDA was prepared in 0.9% sterile saline with 0.02% ascorbic acid; Millipore Sigma, Burlington, MA, United States) and was prepared fresh every 2 h. Using a microinjector (Stoelting Quintessential Stereotaxic Injector Model 53311, Stoelting Co., Wood Dale, IL, United States) connected to a syringe (10 μl; Hamilton Co., Reno, NV, United States) and needle (26 gauge; Hamilton Co.) 5 μg per coordinate of 6-OHDA was administered unilaterally at two coordinates (in mm) within the striatum: AP 1.6, ML 2.4, DV −4.0, and AP 0.2, ML 3.5, DV −6.4. Animals were given 12.5 mg/kg desipramine HCL in sterile 0.9% normal saline, with 10% dimethyl sulfoxide (DMSO; Sigma-Aldrich, St. Louis, MO) prior to delivery of 6-OHDA to prevent noradrenergic neuron damage.

#### 2.3.4 Amphetamine-Induced Rotations

In order to pharmacologically assess the degree of dopaminergic (DA) depletion in the lesioned side, animals were given amphetamine, an indirectly acting DA agonist and placed into a plexiglass cylinder (38 cm tall × 38 cm across) enclosed in an open field box (Yue et al., 2011). Dextro-amphetamine in sterile 0.9% saline (Sigma-Aldrich) was injected i.p. at 5 mg/kg, and rotations were recorded from above using Logitech HD web cameras connected to an Acer Aspire SWS-015 computer. An observer blinded to the groups counted rotations in both the ipsilateral and contralateral rotations for the entire 70 min period. Total number of rotations is reported. Combined number of rotations at 2- and 4-weeks testing are represented as cumulative rotations. The observed contralateral rotational behavior in the vehicle-treated animals in Study 1 and in some animals of Study 2 is indicative of a very mild lesion (<30% loss) and likely due to the denervation of DA neurons and subsequent hypersensitivity of the DA receptors in the lesioned area of the striatum, as shown by others (Carey, 1992; Robinet and Bardo, 2001). This is in contrast to the more commonly used more severe unilateral lesions (>40%), leading to ipsilateral rotations (Björklund and Dunnett, 2019).

#### 2.3.5 Immunohistochemistry

24 h after completion of the 4 weeks behavioral tasks, rats were transcardially perfused using sterile 0.9% normal saline, followed by 4% paraformaldehyde (PFA). Whole brains were put in 4% PFA for 24 h, at which point they were moved to 30% sucrose solution. 40 μm sections were obtained via cryosectioning of whole brains on a cryostat (Leica CM 1850). A 0.3% H_2_0_2_ solution was used to block endogenous peroxidases, and antigen retrieval was performed on free floating sections using heated 0.1 M citrate buffer (pH 6.0) and then were blocked using Bloxall (Vector Labs, Burlingame, CA). Sections were then incubated with the primary antibodies overnight at 4°C for 24 h (TH, AB152, 1:4,000, Millipore-Sigma). After washing in TBS tissue TH+ cell visualization was achieved by incubating for 30 min in Impress AP polymer detection kit, followed by chromogenic reaction with Nova Red Substrate kit (MP-5402, and SK-4800 Vector Labs, Burlingame, CA). Tissue was mounted to subbed slides and cover slipped with Cytoseal 60 mounting medium.

#### 2.3.6 Unbiased Stereology

Stereological probes were applied using a Zeiss Imager M2 microscope (Carl Zeiss, Jena, Germany) equipped with StereoInvestigator software (MBF Bioscience, VT, United States) according to previously published methods (Corenblum et al., 2015; Anandhan et al., 2021). Using the optical fractionator probe, cells were counted under the ×63 oil immersion objective by a blinded observer. TH+ cells were counted in sections 480 μm apart using a grid size of 170 × 100 μm and counting frame size of 75 × 75 μm. The Gundersen method for calculating the coefficient of error was used to estimate the accuracy of the optical fractionator results. Coefficients obtained were less than 0.1.

#### 2.3.7 Microglia Analysis

Three coronal brain sections that contained the substantia nigra (SN) were randomly chosen for each animal. For immunofluorescence of microglia free-floating brain sections were first blocked in 10% horse serum (Vector Laboratories, S-2000–20, Burlingame, CA) and buffer solution (0.01 M PBS, 0.05% Triton, and 0.04% NaN_3_) for 1 h followed by a 72 h incubation with primary antibodies as appropriate: rabbit anti-IBA1 at 1:1,000 (Wako, 019–19741, Madison, WI), mouse anti-TH at 1:2000 (R& D Systems, MAB7566, Minneapolis, MN). Tissue was incubated in secondary antibodies (Jackson ImmunoResearch Laboratories, West Grove, PA): donkey anti-rabbit Alexa 488 (711–545-152), Donkey anti-mouse Alexa 594 (715–585-151) at 1:250 for 4 h. All incubation steps were performed at room temperature; washes between incubations were with 0.01 M PBS for 15 min. Vectashield (Vector Laboratories, H-1000) was used to coverslip mounted tissue.

Images were acquired on a confocal microscope (Zeiss NLO 880, San Diego, CA) in 3 fields of view within the SN of each intact and lesioned hemisphere using a ×40 objective (473.33 × 473.33 μm area). Z-stacks were compressed to a 2D image using maximum intensity, channel split, and files saved as. tiff files using the ImageJ software (NIH, v1 53c). Threshold parameters were consistent for all images, and the percent area of IBA1 immunofluorescence was measured for each image. The number of cell somas was counted in the IBA1 channel, an approximation of the number of cells imaged in each frame, and all percent area data was divided by this cell count.

Ramified microglial morphology was quantified using an objective and computer-aided skeleton analysis method previously described in detail (Young and Morrison, 2018). After thresholding, a series of ImageJ plugins were consistently applied across all images to ensure adequate visualization of cell process before the conversion to binary and skeletonized images. The skeletonized representations of original photomicrographs were used for data collection of three morphology parameters using the AnalyzeSkeleton (2D/3D) plugin: summed number of endpoints/frame and summed process length/frame and summed number of branches/frame. All data were then first divided by cell soma counts within the frame to result in a per cell analysis. Sampling averages were calculated for each animal within each region (intact SN and lesion SN) prior to statistical analyses. All analyses were carried out by researchers blinded to the treatment group.

#### 2.3.8 Statistical Analyses

For the microdialysis experiment a repeated measures Mann-Whitney test was used. For each TBI analysis, shams receiving 2LS80Mel or shams receiving saline were statistically compared. No differences were detected between sham groups, so shams were combined into a single control group. Bout episode duration and differences in rotarod performance were analyzed using a repeated measure two-way analysis of variance (ANOVA) followed by Tukey’s multiple comparisons test. Cumulative sleep (min), novel object recognition, inflammatory cell populations, cytokines, and microglial cell count, endpoints per cell, and process length per cell were analyzed using a one-way ANOVA followed by Tukey’s multiple comparisons test. Non-parametric NSS data were analyzed using a Friedman repeated measure ANOVA, followed by Dunn’s multiple comparisons test. For all data, the assumption that variables were normally distributed was verified. Resulting critical values are included in the Results. All normally distributed data were screened using the Grubb’s outlier test for statistical outliers, outliers are reported in the Results. For the PD analysis, differences in vehicle vs. treatment groups in amphetamine-induced rotational behavior were evaluated with unpaired two-tailed student *t*-tests. For the unbiased stereology in intact vs. lesioned hemispheres in each group in study 1 we utilized a one-way ANOVA with Šídák’s multiple comparisons tests. For study 2, one-way ANOVA with Holm-Šídák’s multiple comparisons tests was used to compare multiple groups in stereology and morphometric analyses. All of the aforementioned analyses were conducted, and associated figures were constructed, using GraphPad Prism 9; results are shown as mean ± SEM, with statistical significance assigned at the 95% confidence level (α < 0.05).

## 3 RESULTS

### 3.1 Synthesis

A modest library of 47 glycopeptide analogues of PACAP were successfully synthesized, purified, and characterized by HPLC and MS. The carbohydrate moieties were introduced “pre-translationally” by incorporation of Fmoc-Serine glycoside building blocks that were prepared using “minimally competent” InBr_3_ catalysis (Mitchell et al., 2001; Lefever et al., 2012). PACAP is considered a “difficult peptide sequence” due to its length and presence of two dipeptide motifs within its structure that are prone to aspartimide formation (Dölling et al., 1994; Lauer et al., 1995; Subirós-Funosas et al., 2011; Paradís-Bas et al., 2016; Samson et al., 2019). Thus, the use of a single coupling protocol and standard Fmoc amino acid building blocks typically results in low yields and purity. We addressed these problems by utilizing several different coupling protocols for the PACAP derivatives (Figure 4). The glycosidic amino acid at the C-terminus was coupled to the resin using 6-Cl-HOBt and an equimolar amount of DIC in NMP. The residues Tyr^10^ through Leu^27^ were coupled using a standard HBTU/N-Methylmorpholine protocol. Aspartimide formation was suppressed by incorporating Fmoc-protected diamino acid building blocks containing either pseudoproline or dimethoxybenzyl (DMB)-containing motifs (Figure 4) (Haack and Mutter, 1992; Wöhr and Mutter, 1995; Wöhr et al., 1996; Sampson et al., 1999; White et al., 2004; Cardona et al., 2008). The dipeptides Asp^8^-Ser^9^ and Asp^3^-Gly^4^ were coupled using 6-Cl-HOBt/DIC in NMP. The lipophilic tripeptide motif consisting of Ile^5^, Phe^6^, and Thr^7^ residues were coupled using HATU and 2,4,6-collidine in DMF. The remaining amino acids (His^1^ and Ser^2^) were coupled using the 6-Cl-HOBt/DIC protocol. Acetate protection on the carbohydrate hydroxyls were cleanly removed on-resin using a 50% solution of NH_2_NH_2_•H_2_O in NMP. At this stage the peptides were cleaved from the resin with a standard cleavage cocktail [TFA/DCM/H_2_O/HSiEt_3_/anisole (90:10:2:3: 0.5)], precipitated in cold diethyl ether, and purified using RP-HPLC (see section 2). Satisfyingly, this approach resulted in improved purity and yield compared to our initial studies.

### 3.2 *In Vitro* Functional Activity

The *in vitro* functional activity of our glycopeptide analogues was evaluated by measuring their ability to induce cAMP production in three CHO cell lines *individually* expressing the PAC1, VPAC1, or VPAC2 receptors. Not all GPCRs have a full suite of agonists and antagonists to facilitate their study, and these Class B GPCRs are even more challenging in this regard.^2^ Of the 69 unique agonist- or antagonist-bound GCPR structures published between 2000 and 2019, 33 had only antagonists, 11 had only agonists, and 25 had both agonist- and antagonist-bound structures.^3^ Truncated PACAP_6_–_38_ is widely accepted as a PAC1 antagonist,^4^ but the initial studies were done in *tissue,* which may not reflect direct antagonism at the receptor. In any case, we were unable to demonstrate antagonism of PAC1 using PACAP_6_–_38_.

The PACAP glycopeptide analogues were explored *via* 4 different series of structures based on the particular set of substitutions being examined. More specifically, structural modifications in the 1st series of PACAP glycopeptide analogues include stabilizing Leu^17^ and D-Ser^2^ substitutions, but more importantly the main goal was to probe the effects on potency and efficacy upon introduction of various mono- and disaccharides. Subsequently, we explored the effects of shifting the position of the carbohydrate-bearing amino acid at the C-terminus in the 2nd series of PACAP glycopeptide analogues. Additional substitutions that we investigated include Leu^17^→Nva^17^ (Norvaline), L-Thr^2^ or D-Thr^2^ in place of Ser^2^, and Thr^7^→Ala^7^ to obtain PAC1/VPAC1 selective agonists (Bourgault et al., 2009b). The last SAR consideration in the 2nd series of analogues involved examination of the disaccharide-containing analogues’ functional activity at the VPAC1 and VPAC2 receptors. The D-Ser^2^ substitution was held constant in many analogues to examine effects on receptor selectivity. Modifications in the 3rd series of PACAP glycopeptides focused on the importance of the “hinge region” at positions 4 and 5 (Gly and Ile, respectively) in modulation of receptor selectivity. In the 4th series of PACAP glycopeptides we further investigated the effects of “hinge region” substitutions and N-terminal acylation on stability and functional activity, and we reinvestigated the importance of the identity of the carbohydrate residue present at the C-terminus.

#### 3.2.1 1^st^ Series PACAP Glycopeptides

Initial investigations in our PACAP glycopeptide SAR involved the simple introduction of carbohydrate-containing amino acids at the C-terminus (**2LS80Gluc, 2LS280Cel, 2LS80Lact**). Replacement of Met^17^ with Leu (**2LS72–2**), which is not prone to oxidation, and a D-Ser^2^ substitution (**2LS72–3**) were investigated to further improve stability compared to native PACAP_1–27_. Introduction of a carbohydrate did not lead to significant reductions in efficacy or potency (Table 3). These initial results indicate that placement of the carbohydrate residue at the C-terminus does not interfere with receptor binding and activation, which is consistent with observations from other SAR campaigns investigating glycosylation of opioid peptides (Apostol et al., 2020). Furthermore, the D-Ser^2^ and Leu^17^ substitutions were also well tolerated and only lead to minimal reductions in potency and efficacy. This demonstrates that Leu^17^ does not disrupt the α-helix required for receptor binding, and the D-Ser^2^ substitution preserves the N-terminal bioactive conformation necessary for receptor activation.

#### 3.2.2 2^nd^ Series PACAP Glycopeptides

The encouraging *in vitro* results from the 1^st^ series of PACAP glycopeptides prompted us to determine the ideal position of the carbohydrate-containing amino acid in the C-terminus (**CRA3000-CRA3005**). We found that shifting the carbohydrate-bearing amino acid one or two positions over from the C-terminal end led to slight and consistent decreases in potency at each receptor. These decreases were more pronounced in the analogues containing the Ala^7^ substitution (**CRA3003-CRA3005**), which were devoid of activity. Interestingly [D-Ser^2^, Nva^17^], PACAP glucosides (**CRA3000-CRA3005**) were selective for the VPAC1 receptor and exhibited slightly reduced potency at PAC1 and significantly reduced potency at VPAC2 (Table 4). The disaccharide-containing compounds **2LS98Lac, 2LS98Cel**, and **2LS98Mel** were found to be PAC1/VPAC1 selective agonists with reduced potency at VPAC2, but not to the same degree as **CRA3000-CRA3005**. Interestingly, the melibiose-containing analogue (**2LS98Mel**), had enhanced potency at the VPAC2 receptor compared to the compounds containing either lactose (**2LS98Lac**) or cellobiose (**2LS98Cel**) residues. The reason for this enhanced activity at VPAC2 is unknown. In one unrelated case where position 2 and the C-terminus were not being investigated, we prepared **Ac-2LS132**, an N-terminally acylated version of **2LS80Lact. Ac-2LS132** exhibited equipotent activity at PAC1 and VPAC1 and enhanced selectivity at VPAC2 compared to the analogue lacking an N-acyl group, which prompted us to further investigate N-acylation in the 4^th^ series of PACAP glycopeptides, which will be discussed in section 3.2.4.

#### 3.2.3 3^rd^ Series PACAP Glycopeptides

**FIGURE 5 |:**
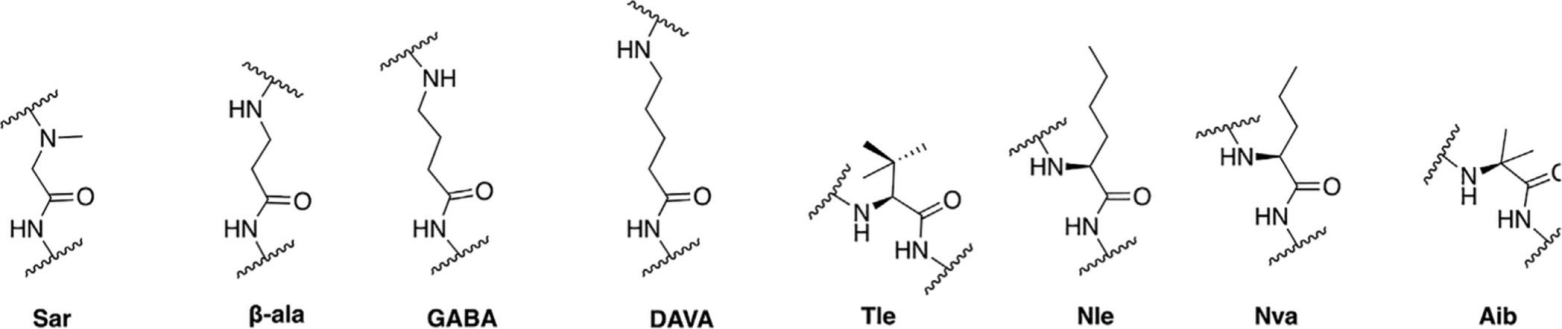
Structures of non-natural amino acids used in these studies.

PACAP_1–27_ and VIP share high structural similarity (68%), especially at the N-terminus. However, the amino acids in the 4^th^ and 5^th^ positions differ between the two peptides. In PACAP, positions 4 and 5 are occupied by Gly-Ile, whereas in VIP they are occupied by Ala-Val (Figure 3). Gly is a known β-turn inducer, while Ala promotes α-helix formation. Furthermore, Ile and Val both contain branched alkyl side chains, but their steric profiles are subtly different. These differences encouraged us to explore the effects of strategic “hinge region” modifications on receptor selectivity. Satisfyingly, *in vitro* functional activity data obtained from the 3^rd^ series PACAP glycopeptides demonstrated that we can successfully fine-tune receptor selectivity with the appropriate hinge region substitutions (Table 4). Substitutions in the 4^th^ position containing turn-inducing motifs (GABA, Sar, β-Ala, DAVA) (Figure 5) were relatively more selective for the PAC1 receptor, with **CRA3006 (GABA)** being an exception. However, compounds containing the linker-like amino acids exhibited reduced activity at all receptor subtypes. **CRA3007 (Sar^4^)**, was ∼5-fold more selective for PAC1 over VPAC1 and ∼24-fold selective for PAC1 over VPAC2. This is an interesting result considering previously reported analogues containing a Sar^4^ substitution were found to be antagonists (Bourgault et al., 2009b). It is possible that the combination of D-Ser^2^ and Sar^4^ induces a bioactive conformation that favors agonist activity. Compounds containing the α-helix-promoting amino acid substitutions Ala^4^ (**CRA3010**) and Aib^4^ (**CRA3011**) were more selective towards the VPAC1 receptor, with CRA3010 exhibiting ∼18-fold selectivity for VPAC1 over PAC1 and CRA3011 exhibiting ∼23-fold selectivity for VPAC1 over PAC1. It was also determined that subtle changes in steric bulk of amino acids in position 5 can also affect receptor selectivity, albeit to a lesser extent than the 4^th^ position modified compounds (Figure 5). For example, **CRA3012-CRA3015** (Val^5^, Leu^5^, Ala^5^, Tle^5^) contain amino acids with bulky side chains, with Ala being an exception. In this series, there were reductions in potency and efficacy at all investigated receptors with no clear trends in receptor selectivity. However, **CRA3013** (Leu^5^) is an interesting case because it was relatively more selective for VPAC1 with an EC_50_ of 20.9 nM. **CRA3016** (Nva^5^) and **CRA3017** (Nle^5^), which contain amino acids with linear alkyl side chains, exhibited significantly improved functional activity at all the examined receptor subtypes. **CRA3017** exhibited the best functional activity of all the analogues in this series. Overall, our strategic hinge region substitutions demonstrate that relatively predictable receptor selectivity profiles can be attained with the appropriate modifications that consider the conformational preference of the amino acid in the 4^th^ position and by the steric profile of the amino acid in the 5^th^ position.

#### 3.2.4 4^th^ Series PACAP Glycopeptides

**FIGURE 6 |:**
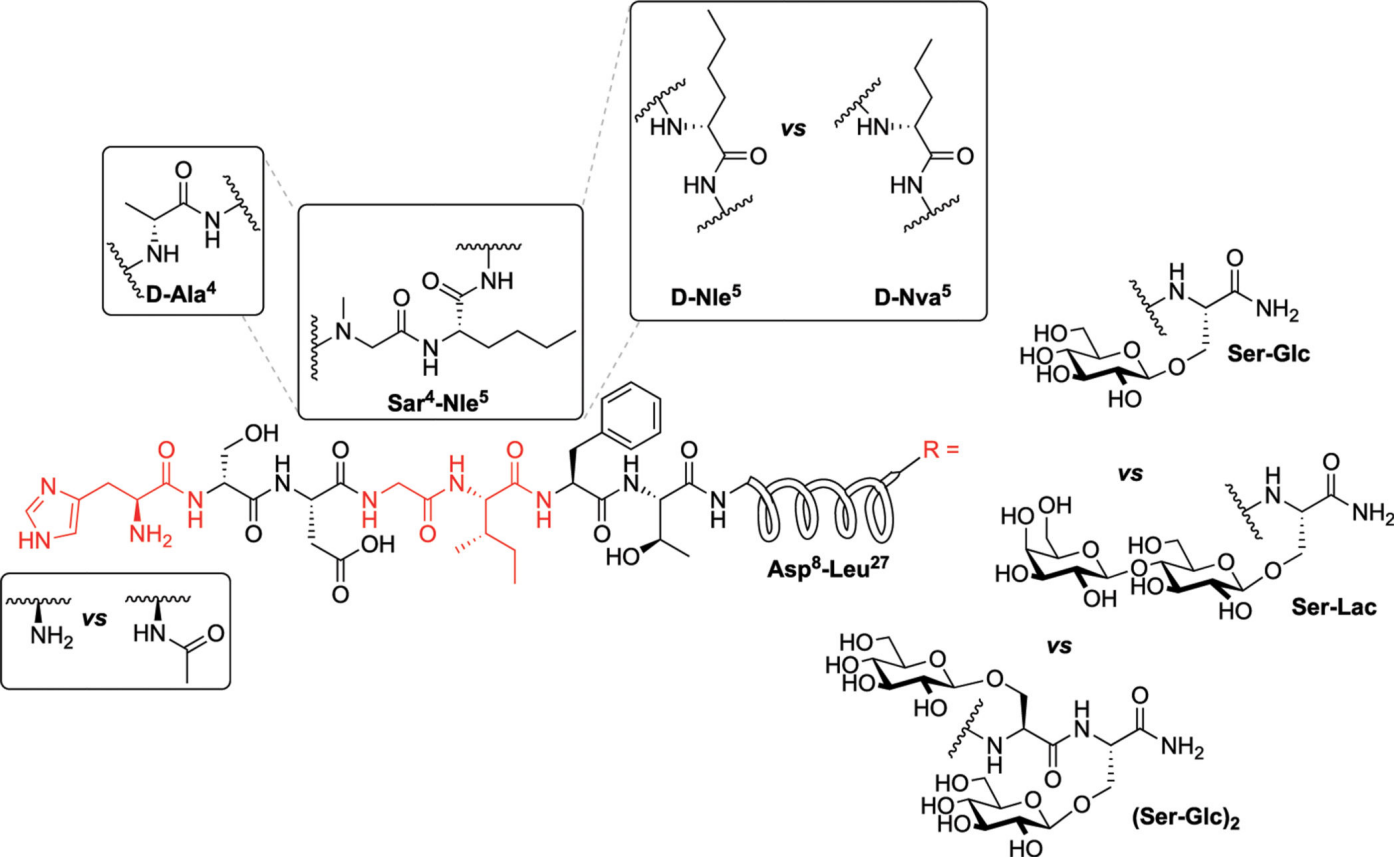
Additional modification of the Hinge region and C-terminus: 4^th^ Series of PACAP Glycopeptides. The identities of the carbohydrate residues at the C-terminus were re-investigated for their effects on potency, efficacy, and receptor selectivity. The hinge region was further explored for the effects of D-amino acids in the 4^th^ and 5^th^ positions on receptor selectivity, and a compound containing both Sar^4^ and Nle^5^ substitutions was also prepared and evaluated. Last, N-terminal acylation was further explored to determine how the acyl group affects receptor selectivity.

In the 4^th^ series of PACAP glycopeptides we further explored substitutions in the hinge region, using *in vitro* functional activity data for the 3^rd^ series PACAP glycopeptides as a guide, and N-acylation at His^1^ (**Ac-2LS132**) (Figure 5). More specifically, analogues containing D amino acid substitutions in positions 4 or 5 were implemented, and an analogue containing both Sar^4^ and Nle^5^ substitutions was also prepared (**CRA3018**). A corresponding N-Acylated compound was prepared for each analogue in this series. Additionally, we reexamined the importance of specific C-terminal carbohydrates with regards to receptor binding, activation, and selectivity. To this end, a lactose-containing analogue of **CRA3000 (CRA3000-Lac)** was prepared, and an analogue of **2LS80Glc** containing two serine glucoside residues was also synthesized (**2LS140**) (Figure 6). It was found that N-terminal acylation led to enhanced selectivity VPAC2, although selectivity towards VPAC1 was observed in some cases (Table 4). This is in line with previous studies demonstrating that N-acyl-His^1^ PACAP27 was more potent at stimulating cAMP production through VIP-like receptors in SUP-T1 cell membranes (Gourlet et al., 1991). Furthermore, it has been previously reported that lipidated analogues of Nle^17^ VIP exhibited equipotent functional activity at the VPAC1/VPAC2 receptors and robust *in vitro* and *in vivo* efficacy in models of neuroprotection and male impotence (Gozes and Fridkin, 1993; Gozes et al., 1994; Gozes et al., 1995; Gourlet et al., 1998). Although it is clear that N-terminal acylation leads to increased activity at the VPAC1/VPAC2 receptors, the molecular and conformational basis for this shift in selectivity is currently unknown. Introduction of D-Nva (**CRA3020**) or D-Nle (**CRA3019**) in the 5^th^ position resulted in drastic reductions in functional activity, indicating that D-amino acids are not well-tolerated in this position. This is consistent with previous SAR studies wherein D-Ile^5^-containing analogues exhibited significantly reduced activity, highlighting the importance of the side chain orientation of Ile^5^ in native PACAP (Bourgault et al., 2009b). Furthermore, it has been suggested that hydrophobic interactions between Ile^5^, Phe^6^, and Thr^7^ are critical for receptor activation (Bourgault et al., 2011). Therefore, it is plausible that introducing D-amino acids in the 5^th^ position, or in the 6^th^ and 7^th^ positions as well, may disrupt these critical hydrophobic interactions and subsequently disfavor PACAP’s ideal bioactive conformation. Unfortunately, **CRA3018**, which contains both Sar^4^ and Nle^5^ substitutions, exhibited significant *decreases* in potency and efficacy at PAC1, VPAC1, and VPAC2. It is likely that this combination led to an unfavorable N-terminal conformation. Interestingly CRA3021, which contains a D-Ala^4^ substitution, exhibited equipotent activity at PAC1 and VPAC1 and significantly reduced selectivity at VPAC2. Despite this, CRA3021 was much less potent compared to native **PACAP**_1–27_. Both **CRA3000Lac** and **Ac-CRA3000Lac** showed significantly improved functional activity compared to the serine glucoside-containing compounds **CRA3000** and **Ac-CRA3000**, further confirming that analogues with a disaccharide exhibit superior activity. **2LS140**, which contains a di-serine glucoside motif, displayed the best functional activity in the 4^th^ series of compounds. Additionally, the functional activity profile of **2LS140** was strikingly similar to that of **2LS80Lact**, which suggests that two serine glucosides can be used as a disaccharide mimic. Thus, we are interested in using **2LS140** as a template for a future series of PACAP glycopeptides to confirm that a di-serine glucoside motif is an appropriate substitution for the serine-lactoside moiety.

### 3.3 *In Vitro* Stability

Initial investigations into the stability of select 2^nd^ series PACAP glycopeptides were carried out in water and in artificial cerebrospinal fluid (aCSF). The compounds remained stable in water, and they exhibited half-life values ranging between 10–15 min in aCSF). Selected 2LS-glycosides, namely the glucoside and lactoside, were examined for their stability in rat serum at 37°C. Upon introduction of a glucose residue (**2LS72–4**) the half-life was extended by approximately 15 min compared to the non-glycosylated compound. The lactose-containing glycopeptide **2LS80Lac** exhibited a half-life extending greater than 1 h, suggesting that disaccharides provide a greater degree of protection from enzymatic degradation compared to a monosaccharide. Selected compounds from the 3^rd^ series of PACAP glycopeptides were subjected to *in vitro* stability studies in rat serum at physiological temperatures as well [(Liu et al., 2222) unpublished results].

### 3.4 *In Vivo* Stability and BBB Penetration

**FIGURE 7 |:**
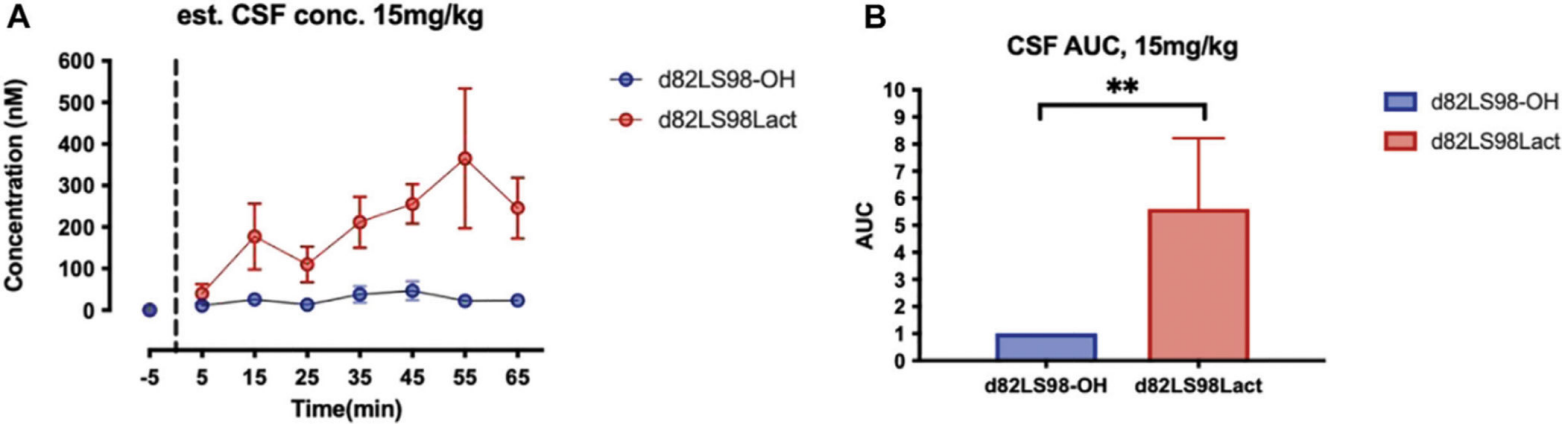
Summary of *in vivo* stability and BBB transport. **(A)** *In vivo* CSF concentrations of d82L98-OH and d82LS98Lact following 15 mg/kg i.v. injection in rats (*n* = 5). Glycopeptide concentrations were quantified by HPLC-MS. **(B)** Area under the curve (AUC) of d82L98-OH and d82LS98Lact following 15 mg/kg i.v. injection in rats. AUCs are normalized to d82L98-OH. d82L98-OH and d82LS98Lact are deuterated mass-shifted analogues of 2L98-OH and 2LS98Lact. Dosage of 15 mg/kg are used to overcome the high detection of limit in CSF. Glycosylated analogues exhibited enhanced BBB transportation with a disaccharide moiety. Concentrations and AUCs are reported by mean ± SEM. Asterisks demote statistical significance using repeated measures Mann-Whitney test, *p* < 0.01.

The *d*8 mass shifted analogues of 2LS72–2 and 2LS80Lac (d82LS98-OH and d82LS98Lact, respectively) were investigated for their *in vivo* BBB transport in rats. More specifically, a technique known as shotgun microdialysis, wherein multiple compounds are injected at one time in a single rat, was utilized to reduce the total number of animals used in the study and to mitigate subtle variations in the injection site (Mabrouk et al., 2012; Hay et al., 2019). Following *i.v.* injection (15 mg/kg) of d82LS98-OH and d82LS98Lact, aliquots of CSF were removed at specific time points and the concentrations of the peptides were quantified by LC-MS. A dosage of 15 mg/kg was selected due to the high detection limit in the CSF. The concentration of the un-glycosylated compound d82LS98-OH was exceptionally low, suggesting poor penetration of the BBB. However, the CSF concentration of the lactose-containing d82LS98Lact was determined to be much higher, peaking around 400 nM (Figure 7A). AUC analysis of d82LS98-OH and d82LS98Lact CSF concentrations (normalized to d82LS98-OH) demonstrate the difference in BBB penetration of the two compounds (Figure 7B). These results strongly indicate that the presence of a carbohydrate motif significantly enhances the penetration of glycopeptides across the BBB compared to their non-glycosylated counterparts. Unlike previous glycopeptides investigated in our lab, native PACAP itself is able to penetrate the BBB to a limited extent. Specifically, PACAP_1–38_ passes through the BBB via the PTS-6 transporter, while PACAP_1–27_ is hypothesized to penetrate *via* a non-saturable diffusion mechanism, albeit at a lower efficiency compared to PACAP_1–38_, since no such PACAP_1–27_ selective transporter is known to exist (Banks et al., 1993).

### 3.5 *In Vivo* Models of Neuroprotection and Anti-Inflammatory Activity: Preliminary Success in TBI and PD Animal Models

Following extensive *in vitro* and *in vivo* experiments assessing the functional activity and stability and BBB transport of our PACAP glycopeptides, two compounds were chosen for assessment in animal models of TBI and PD. More specifically, 2LS80Mel was chosen for evaluation in a rodent model of TBI, and 2LS98Lac was examined for its neuroprotective potential in the progressive 6-hydroxydopamine (6-OHDA) hemi-parkinsonian rat model.

#### 3.5.1 *In Vivo* TBI

**FIGURE 8 |:**
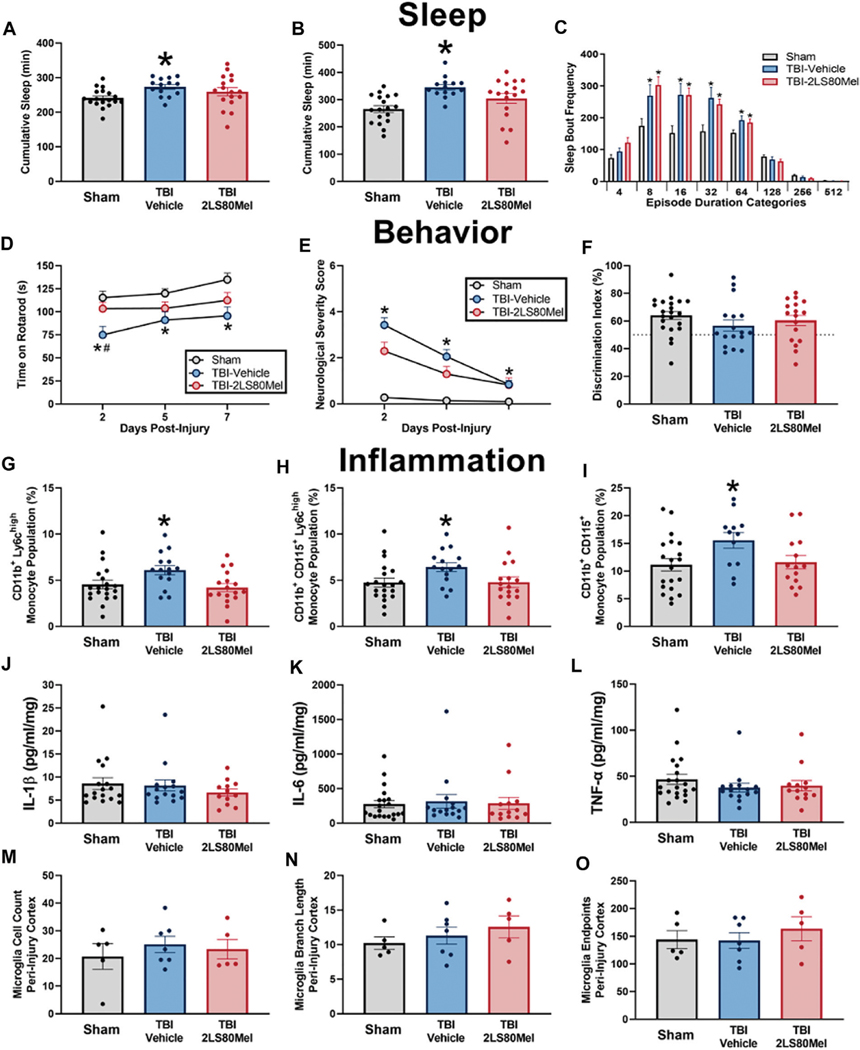
Summary of protective and anti-inflammatory effects of 2LS80Mel in a mouse model of TBI. Mice were subjected to diffuse TBI or control sham surgery and treated with either 2LS80Mel or sterile saline. The *in vivo* efficacy of 2LS80Mel was then evaluated by assessing its effects on the sleep-wake behavior **(A–C)**, neurological and motor skill deficits **(D–F)**, monocyte populations **(G–I)**, concentrations of inflammatory cytokines **(J–L)**, and microglial morphology **(M–O)**. Overall, 2LS80Mel attenuated behavioral, neurological, and motor skill deficits. Furthermore, 2LS80Mel prevented increases in peripheral monocyte populations. There were no significant differences in inflammatory cytokine concentrations or microglial ramification between the 2LS80Mel-treated, untreated, and sham animals.

Lead compound **2LS80Mel** was evaluated for its neuroprotective potential *in vivo* utilizing a mouse model of traumatic brain injury (TBI). Mice were pretreated with either a single dose of 2LS80Mel (10 mg/kg *i.p.*) or sterile saline 30 min prior to being subjected to a midline fluid percussion injury. The mice were then evaluated for sleep-wake behavior (Figures 8A–C), motor skills and cognition, (Figures 8D–F), the presence of inflammatory markers, (Figures 8G–L), and microglial morphology (Figures 8M–O).

Rowe and others have previously reported diffuse TBI increases post-traumatic sleep immediately following injury, and attenuation of TBI-induced sleep using pharmacological therapies improved outcomes (Rowe et al., 2014c; Rowe et al., 2018; Rowe et al., 2019). In this study, diffuse TBI led to an increase in cumulative minutes slept in the first 6 h post-injury, and mice administered **2LS80Mel** prior to TBI slept comparable minutes to uninjured sham animals (F_2,46_ = 3.364, *p =* 0.0433; Figure 8A). Similarly, TBI led to an increase in sleep in the first dark period, when mice are typically awake, and 2LS80Mel prevented this injury-induced increase in sleep (F_2,46_ = 7.710, *p =* 0.0013; Figure 8B). It has been demonstrated that brain-injured mice sleep more but have shorter, fragmented bouts of sleep, so we analyzed sleep fragmentation as previously published (Rowe et al., 2019; Giordano et al., 2020). The frequency of individual sleep bouts with different episode durations was analyzed and all brain-injured mice had more short bouts (<∼1 min) compared to uninjured shams (F_2,46_ = 9.301, *p =* 0.0004; Figure 8C).

To asses motor function, the Rotarod test was used as previously published by Rowe and others (Rowe et al., 2014b; Rowe et al., 2018). Motor function was tested as the latency to stay on the Rotarod over 7 DPI, with significant effects of both time post-injury (F_2,110_ = 8.941, *p* = 0.0003; Figure 8D) and between treatment groups (F_2,53_ = 7.876, *p =* 0.001). Tukey’s *post hoc* analysis indicated that compared to uninjured shams, TBI-vehicle mice had significantly shorter latencies to fall from the rod at 2, 5, and 7 DPI. **2LS80Mel** prevented this motor deficit, and TBI-**2LS80Mel** mice had latencies to fall from the rod that were comparable to uninjured shams at all time points post-injury. Diffuse TBI also resulted in gross neurological dysfunction evaluated by the modified NSS, as we have previously published (Rowe et al., 2014b; Rowe et al., 2018). TBI-vehicle mice had sensorimotor deficits at all time points compared to shams, indicated by a high NNS score. These deficits were prevented by the administration of **2LS80Mel** (F_r_ = 6.0, *p =* 0.028; Figure 8E). There were no TBI-induced cognitive deficits among any group measured by the NOR task (F_2,53_ = 1.129, *p =* 0.331; Figure 8F). Overall, **2LS80Mel** successfully attenuated motor skill and cognitive deficits induced by TBI.

Analysis of inflammatory markers and microglial morphology in the different groups of mice further confirmed protective/anti-inflammatory activity of **2LS80Mel**. TBI resulted in higher CD11b^+^Ly6C^high^ (F_2,49_ = 4.259, *p =* 0.019; Figure 8G) and CD11b^+^CD115^+^Ly6C^high^ (F_2,49_ = 3.250, *p =* 0.047; Figure 8H) monocyte populations compared to uninjured shams. This TBI-induced increase was not seen in mice treated with **2LS80Mel** prior to injury. There were no differences in neutrophil populations among groups (F_2,49_ = 0.0159, *p =* 0.984; Figure 8I). Measurements of pro-inflammatory cytokines revealed no differences in IL-1β (F_2,41_ = 0.6789, *p* = 0.513; Figure 8), IL-6 (F_2,45_ = 0.0745, *p =* 0.928; Figure 8K), or TNF-α (F_2,45_ = 0.8546, *p =* 0.432; Figure 8L) at 2 DPI among groups. There were also no differences in these peripheral cytokines at 14 DPI (**data not shown**). Microglial ramification was quantified at 15 DPI. Diffuse TBI did not alter microglia cell number (F_2,14_ = 0.3795, *p* = 0.691; Figure 8M), microglia branch length per cell (F_2,14_ = 0.7496, *p* = 0.491; Figure 8N), or microglia endpoints per cell (F_2,14_ = 0.4528, *p =* 0.645; Figure 8O) measured in the peri-injury cortex at 15 DPI. There were no measurable differences in the S1BF or perirhinal cortex at 15 DPI (**data not shown**).

Overall, **2LS80Mel** demonstrated protective effects in a mouse model of diffuse TBI. A single injection of **2LS80Mel** resulted in attenuation of common sensorimotor and motor deficits observed in TBI and TBI-induced post-traumatic sleep. Furthermore, TBI-induced increases in peripheral monocyte populations were mitigated by **2LS80Mel**. Despite no significant differences between **2LS80Mel**-treated and saline-treated groups in levels of select inflammatory cytokines and microglial morphology, **2LS80Mel** still demonstrated robust neuroprotection and anti-inflammatory activity *in vivo*.

#### 3.5.2 *In Vivo* Parkinson’s Disease Model

**FIGURE 9 |:**
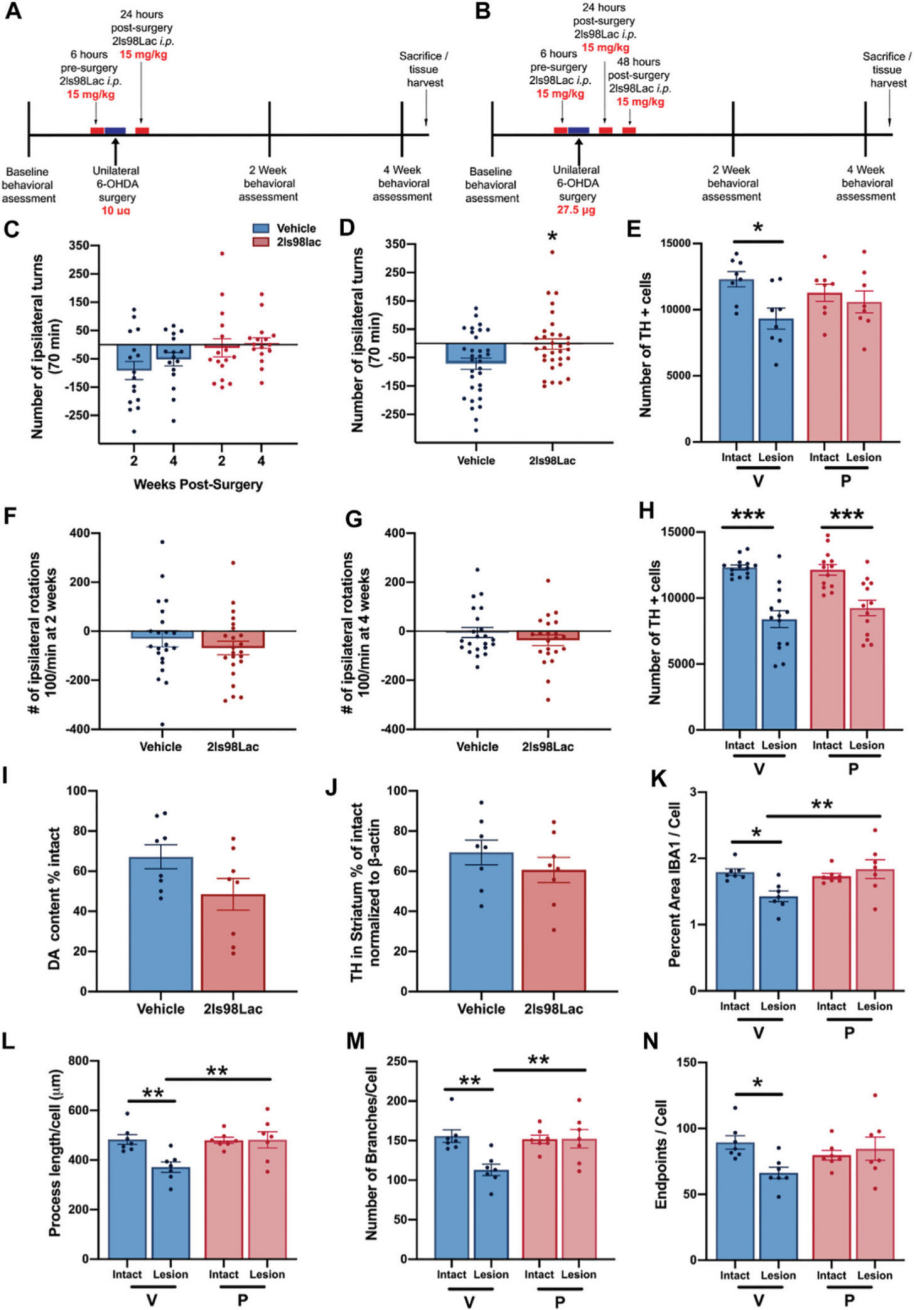
Evaluation of neuroprotection of the PACAP glycopeptide 2LS98Lac in rat Parkinson’s disease models. **(A)** Scheme for study 1, using a mild PD lesion. **(B)** Scheme for study 2, using a moderate PD lesion. **(C)** Systemic injection (*i.p*.) of 2LS98Lac reduces 6-OHDA-induced lesion damage in the mild 6-OHDA hemi-parkinsonian rat model. Amphetamine-induced rotations at 2- and 4-weeks post-lesion are plotted (mean rotations ± SEM). **(D)** The mean ± SEM cumulative amphetamine-induced rotations are plotted showing that treatment with the PACAP glycopeptide reduced the number of rotations indicative of a protective effect. *p < 0.05. **(E)** Unbiased stereology of TH-positive dopaminergic neurons in the substantia nigra (SNc) in study 1 reveal a significant 6-OHDA-induced loss of TH-positive neurons on the lesioned side in the vehicle control group (V), but not the 2LS98Lac-treated group (P). *p < 0.05. **(F,G)** Systemic injection (*i.p*.) of 2LS98Lac does not reduce 6-OHDA-induced lesion damage in the moderate 6-OHDA hemi-parkinsonian rat model. Amphetamine-induced rotations at 2 **(F)** and 4 **(G)** weeks post-lesion are plotted (mean rotations ± SEM). **(H)** Unbiased stereology of TH-positive neurons in the SNc in study 2 reveal a significant 6-OHDA-induced loss of TH-positive neurons on the lesioned side for both groups (V and P), but no group difference. ***p < 0.001. **(I)** 2LS98lac-treatment did not change mean (±SEM) striatal dopaminergic content (DA) analyzed with HPLC-EC. Mean data (±SEM) are plotted as % control. **(J)** 2LS98lac-treatment did not change striatal TH expression, quantified with semi-quantitative western analysis with beta-actin (βA) as internal standard. Mean data (±SEM) are plotted as % control. **(K–N)** 2LS98lac rescues 6-OHDA induced morphological changes to microglia (mean data ± SEM). Specifically, we show%area IBA1/cell **(K)**, process length/cell **(L)**, number of branches/cell **(M)** and endpoints/cell **(N)**. *p < 0.05; **p < 0.01.

The classical mild progressive 6-OHDA hemi-parkinsonian rat model was used to assess the neuroprotective potential of **2LS98Lac** (Figures 9A,B). Briefly, the rats were given unilateral lesions by treatment with 6-OHDA. Then, the rats were challenged with a dopaminergic receptor agonist (amphetamine in our case) and assessed for their turning behavior (Kelly et al., 1975; Torres and Dunnett, 2007; Da Cunha et al., 2008). The observed rotational behavior is likely due to the denervation of dopaminergic neurons and subsequent hypersensitivity of the dopaminergic receptors in the lesioned area of the brain. In the first experiment, mild lesions (25%) were induced (Figure 9A) to simulate the early stages of PD, and amphetamine was used to induce contralateral rotations. Rats were treated with either vehicle or **2LS98Lac** (15 mg/kg, *i.p.*) 6 h prior to lesioning and 24 h post-lesion. Excitingly, the total number of cumulative contralateral rotations significantly decreased (*t =* 2.55; *p =* 0.013) in the **2LS98Lac**-treated rats compared to vehicle-treated rats (Figure 9C and Figure 9D). Following behavioral analysis, the number of tyrosine hydroxylase (TH)-positive neurons in the SNpc of both vehicle-treated and **2LS98Lac**-treated animals was determined using immunohistochemical staining and unbiased stereology. While there was a significant reduction of TH-positive cells in the lesioned vs. the intact side in vehicle-treated animals, indicating successful lesioning (F_3,28_ = 3.024, *p =* 0.0199), the lack of such a difference in the **2LS98Lac**-treated rats indicates that neuronal cell death was attenuated by **2LS98Lac** treatment (Figure 9E). Both behavioral and histological data obtained from these experiments indicate that **2LS98Lac** successfully elicited neuroprotection.

In a follow-up study, we probed the neuroprotective potential of **2LS98Lac** in rats with more severe (40%) 6-OHDA-induced lesions and three treatment-injections with **2LS98Lac** (Figure 9B). In this study, we did not see a difference in the amphetamine-induced rotations at either 2 weeks (F_21–22_ = 1.385, *p =* 0.3811; Figure 9F), or 4 weeks post-lesion (F_21–21_ = 1.070, *p =* 0.3005; Figure 9G) between vehicle and **2LS98Lac**. While in the nigral TH-cell count a mean increase of 850 cells on the lesioned side in the **2LS98Lac** group vs. the vehicle group (9,244 vs. 8,391) was seen, this proved to be not significant between the lesioned sides of treatment vs. vehicle (F_3–50_ = 17.11, *p =* 0.2208; Figure 9H). There also was no significant difference between treatment and vehicle in striatal TH (F_7–7_ = 1.050, *p =* 0.3362; Figure 9I), as measured with western analysis, or striatal DA content (F_7–7_ = 1.748, *p =* 0.0798; Figure 9J), indicating lack of meaningful protection against the more severe insult.

**FIGURE 10 |:**
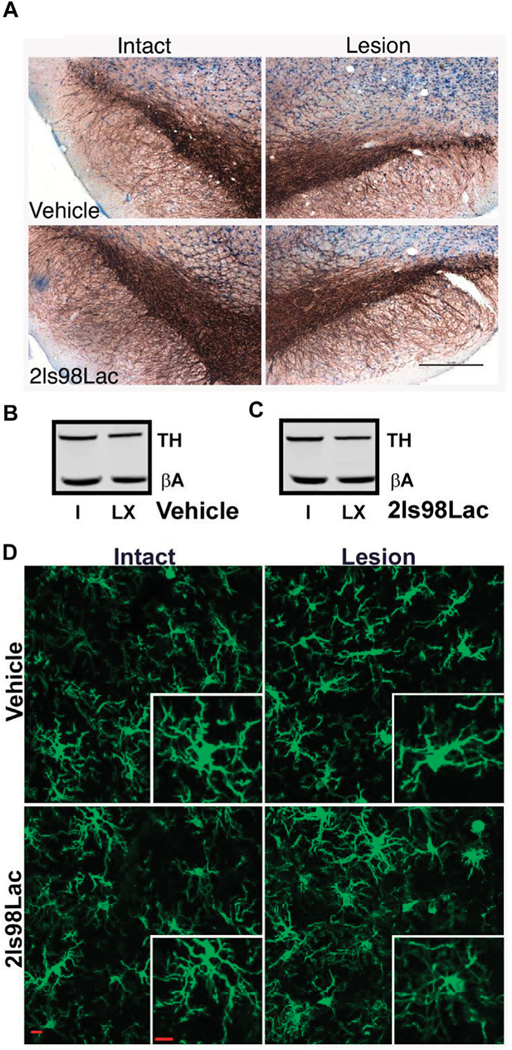
Example images for the analyses of the PACAP glycopeptide 2LS98Lac in the rat Parkinson’s disease model. **(A)** Example images of TH staining in the SNc that had been analyzed with unbiased stereology in Figure 9H to identify dopaminergic neurons within the SNc (40 μm thick serial sections were obtained and sampled every 480 μm. The scale bar is 500 μm. **(B,C)** Example western blots for the semi-quantitative western analysis done in Figure 9J for striatal TH with beta-actin (βA) as internal standard for the vehicle **(B)** and 2LS98Lac **(C)** groups. I: intact hemisphere; LX: lesioned hemisphere. **(D)** Example images for morphological microglia analyses, as done in Figures 9K–N, after confocal imaging of Iba1 stained brain sections (40 μm thick, sampled at 3 regions throughout the SNc). The scale bars are 10 μm.

We also evaluated microglia morphology in this experiment, to investigate effects on neuroinflammation, and found that 6-OHDA-lesioning did lead to a significant change in the morphologic phenotype that was fully reversed by treatment with **2LS98Lac** (Figures 9K–N), indicating modulation of neuroinflammation. Specifically, 6-OHDA did lead to reduction of % area IBA-1/cell (F_3–24_ = 4.463, *p* = 0.0140; Figure 9K), process length/cell (F_3–24_ = 5.925, *p* = 0.0057; Figure 9L), branches/cell (F_3–24_ = 5.845, *p* = 0.0042; Figure 9M), and endpoints/cell (F_3–24_ = 2.943, *p* = 0.0286; Figure 9N) on the lesioned side. Treatment with **2LS98Lac** did lead to an increase of the parameters on the lesioned side back to levels on the intact side. Specifically, there was a significant increase on the lesioned side in the treatment vs. the vehicle group in the % area IBA-1/cell (F_3–24_ = 4.463, *p* = 0.0087; Figure 9K), process length/cell (F_3–24_ = 5.925, *p* = 0.0057; Figure 9L), branches/cell (F_3–24_ = 5.845, *p* = 0.0056; Figure 9M), and a trend toward increase in endpoints/cell (F_3–24_ = 4.463, *p =* 0.0690; Figure 9N). Example images for the nigral IHC for TH and for IBA-1, as well as the semi-quantitative western analyses of striatal TH and beta-actin are presented in Figures 10A–D.

## 4 DISCUSSION

In summary, we have produced a series of brain penetrant PACAP glycopeptide analogues with improved PK/PD properties. Furthermore, preliminary studies in animal models of PD and TBI demonstrate the neuroprotective and anti-inflammatory potential of select PACAP glycopeptides. Introduction of monosaccharides and disaccharides into the backbone of PACAP did not significantly reduce functional activity at the PAC1, VPAC1, or VPAC2 receptors. Disaccharide-containing and di-glucoside PACAP analogues containing lactose or di-glucoside moieties exhibited improved functional activity compared to their monosaccharide counterparts.

Furthermore, the optimal position of the carbohydrate-bearing amino acid was determined to be directly at the C-terminal end. Additional SAR investigations included modifications of the amino acids at positions 2, 4, 5, 7, 17 and N-terminal acylation. Substitution of Met^17^ with either Leu or Nva did not alter the *in vitro* functional activity of the peptides. Reversing the chirality of Ser^2^ sidechain only led to negligible reductions in functional activity. Modifications at positions 4 and 5, otherwise known as the hinge region, demonstrated that receptor selectivity can be successfully fine-tuned with the appropriate substitutions. Amino acids in position 4 favoring β-turn or α-helical conformations result in enhanced selectivity for PAC1 or VPAC1, respectively.

Additionally, introducing amino acids with side chains of varying steric bulk (linear vs. branched) in position 5 can also alter receptor selectivity, albeit to a lesser degree compared to the substitutions made in the 4^th^ position. Additional modifications investigated in the 4^th^ series of PACAP glycopeptides further highlight the sensitivity of the hinge region to drastic conformational changes induced by D-amino acids and *N*-methyl amino acids. N-acylation of the N-terminus led to increased selectivity predominantly at VPAC2, but selectivity was also enhanced at VPAC1 in some cases. This is in line with previous investigations into N-terminally lipidated VIP analogues and could suggest the existence of a hydrophobic region within the VPAC1/VPAC2 receptors’ transmembrane regions, which is not present in PAC1, that can interact with lipophilic moieties (Gourlet et al., 1998).

Results obtained from stability studies both *in vitro* and *in vivo* indicate that our glycosylated PACAP analogues were more robust than their non-glycosylated counterparts. The disaccharide-containing glycopeptides exhibited superior stability compared to glycopeptides containing a single glucose residue, except in cases where a D-Ser^2^ substitution was implemented. Further experiments are required to determine the importance of the hinge region and N-terminal acylation on the stability of our PACAP glycopeptides. *In vivo* BBB transport studies demonstrated that our PACAP glycopeptides do penetrate the BBB with higher efficiency compared to the native peptide. Investigations further probing this observation are currently ongoing.

Most importantly, it was demonstrated that our PACAP glycopeptide analogues, specifically **2LS80Mel** and **2LS98Lac**, were able to elicit neuroprotection in animal models of TBI and a mild-lesion model of PD. Motor skill deficits observed in vehicle-treated animals in both TBI and mild-PD lesion (25% loss) experiments were attenuated in the PACAP glycopeptide-treated groups. Biochemical and immunohistochemical analyses reinforced the behavioral observations, confirming the protective effects exerted by our PACAP glycopeptides. In contrast, the PACAP glycopeptide failed to be protective against a moderate PD lesion (>40% loss), despite clear effects on microglial morphology, indicating still successful modulation of neuroinflammation. The lack of a meaningful therapeutic effect in this moderate PD lesion model could mean that higher levels of drug in the CNS are needed to achieve enough activity to combat more severe insults, or alternatively one could explore the different receptor specificity (PAC1 vs. VPAC1/2) to improve therapeutic potency.

One possibility in the future would be to evaluate a recent series of shorter PACAP glycopeptide analogues that showed varying receptor profiles between PAC1, VPAC1 and VPAC2 (Apostol et al., 2021). Notably, the two compounds were administered *i.p.* in both experiments, demonstrating successful penetration of the BBB. Our compounds are particularly unique in this aspect considering a lack of neuroprotective, regenerative, and BBB permeable therapies on the market for acute and progressive neurodegenerative disorders.

In conclusion, glycosylation has proven to be an effective strategy for making PACAP “druggable”, and further development of our lead compounds may 1 day yield promising therapies for PD, TBI, and other neurodegenerative disorders.

## Supplementary Material

5 mg/Kg AUC Serum

15 mg/Kg CSF AUC

5 mg/Kg Curve Serum

## Figures and Tables

**TABLE 1 |:** Summary of PACAP glycopeptide structures.

Compound	Structure
1st series	

2LS72-1	HSDGIFTDSY_10_SRYRKQMAVK_20_KYLAAVL-CONH_2_
2LS72-2	HSDGIFTDSY_10_SRYRKQLAVK_20_KYLAAVL-CONH_2_
2LS72-3	HsDGIFTDSYioSRYRKQLAVK2oKYLAAVL-CONH2
2LS80Gluc	HSDGIFTDSY_10_SRYRKQLAVK_20_KYLAAVL-Ser(Glc)-CONH_2_
2LS80Lact	HSDGIFTDSY10SRYRKQLAVK20KYLAAVL-Ser(Lact)-CONH2
2LS80Cel	HSDGIFTDSY_10_SRYRKQLAVK_20_KYLAAVL-Ser(Cell)-CONH_2_
2LS80Mel	HSDGIFTDSY_10_SRYRKQLAVK_20_KYLAAVL-Ser(Mel)-CONH_2_

2nd Series	

CRA3000	HsDGIFTDSYioSRYRKQ-Nva-AVK2oKYLAAVL-Ser(Glc)-CONH2
CRA3001	HsDGIFTDSY_10_SRYRKQ-Nva-AVK_20_KYLAAV-Ser(Glc)-CONH_2_
CRA3002	HsDGIFTDSYioSRYRKQ-Nva-AVK2oKYLAA-Ser(Glc)-L-CONH2
CRA3003	HsDGIFADSY_10_SRYRKQ-Nva-AVK_20_KYLAAVL-Ser(Glc)-CONH_2_
CRA3004	HsDGIFADSY_10_SRYRKQ-Nva-AVK_20_KYLAAV-Ser(Glc)-CONH_2_
CRA3005	HsDGIFADSY_10_SRYRKQ-Nva-AVK_20_KYLAA-Ser(Glc)-L-CONH_2_
2LS98Mel	HSDGIFTDSY_10_SRYRKQLAVK_20_KYLAAVL-Ser(Mel)-CONH_2_
2LS98Cell	HSDGIFTDSY_10_SRYRKQI_AVK_20_KYI_AAV_d8_L-Ser(Cell)-CONH_2_
2LS98Lac	HSDGIFTDSY_10_SRYRKQLAV_d8_K_20_KYLAAV_d8_L-Ser(Lact)-CONH_2_

3rd Series	

CRA3006	HsD-GABA-IFTDSY_10_SRYRKQ-Nva-AVK_20_KYLAAVL-Ser(Glc)-CONH_2_
CRA3007	HsD-Sar-IFTDSY_10_SRYRKQ-Nva-AVK_20_KYLAAVL-Ser(Glc)-CONH_2_
CRA3008	HsD-pAla-IFTDSY_10_SRYRKQ-Nva-AVK_20_KYLAAVL-Ser(Glc)-CONH_2_
CRA3009	HsD-DAVA-IFTDSYioSRYRKQ-Nva-AVK2oKYLAAVL-Ser(Glc)-CONH2
CRA3010	HsDAIFTDSY_10_SRYRKQ-Nva-AVK_20_KYLAAVL-Ser(Glc)-CONH_2_
CRA3011	HsD-Aib-IFTDSY SRYRKQ-Nva-AVK2oKYLAAVL-Ser(Glc)-CONH2
CRA3012	HsDGVFTDSY_10_SRYRKQ-Nva-AVK_20_KYLAAVL-Ser(Glc)-CONH_2_
CRA3013	HsDGLFTDSYioSRYRKQ-Nva-AVK2oKYLAAVL-Ser(Glc)-CONH2
CRA3014	HsDGAFTDSY_10_SRYRKQ-Nva-AVK_20_KYLAAVL-Ser(Glc)-CONH_2_
CRA3015	HsDG-Tle-FTDSY_10_SRYRKQ-Nva-AVK_20_KYLAAVL-Ser(Glc)-CONH_2_
CRA3016	HsDG-Nva-FTDSY_10_SRYRKQ-Nva-AVK_20_KYLAAVL-Ser(Glc)-CONH_2_
CRA3017	HsDG-Nle-FTDSY_10_SRYRKQ-Nva-AVK_20_KYLAAVL-Ser(Glc)-CONH_2_
2LS132	HSDGIFTDSYioSRYRKQLAVK2oKYI_AAVLS(l_ac)-CONH2

4th Series	

Ac-2LS132	Ac-HSDGIFTDSY_10_SRYRKQI_AVK_20_KYI_AAVLS(l_ac)-CONH_2_
Ac-CRA3000	Ac-HsDGIFTDSY_10_SRYRKQ-Nva-AVK_20_KYLAAVL-Ser(Glc)-CONH_2_
CRA3000-Lac	HsDGIFTDSY_10_SRYRKQ-Nva-AVK_20_KYLAAVLSer (Lac)-CONH_2_
Ac-CRA3000-Lac	Ac-HsDGIFTDSY_10_SRYRKQ-Nva-AVK_20_KYLAAVLSer (Lac)-CONH_2_
CRA3018	HsD-Sar-Nle-FTDSY_10_SRYRKQ-Nva-AVK_20_KYLAAVL-Ser(Glc)-CONH_2_
Ac-CRA3018	Ac-HsD-Sar-Nle-FTDSY_10_SRYRKQ-Nva-AVK_20_KYLAAVL-Ser(Glc)-CONH_2_
CRA3019	HsDG-D-Nle-FTDSYioSRYRKQ-Nva-AVK2oKYLAAVL-Ser(Glc)-CONH2
Ac-CRA3019	Ac-HsDG-D-Nle-FTDSY_10_SRYRKQ-Nva-AVK_20_KYLAAVL-Ser(Glc)-CONH_2_
CRA3020	HsDG-D-Nva-FTDSY_10_SRYRKQ-Nva-AVK_20_KYLAAVL-Ser(Glc)-CONH_2_
Ac-CRA3020	Ac-HsDG-D-Nva-FTDSY_10_SRYRKQ-Nva-AVK_20_KYLAAVL-Ser(Glc)-CONH_2_
CRA3021	HsDaIFTDSY_10_SRYRKQ-Nva-AVK_20_KYLAAVL-Ser(Glc)-CONH_2_
Ac-CRA3021	Ac-HsDaIFTDSY_10_SRYRKQ-Nva-AVK_20_KYLAAVL-Ser(Glc)-CONH_2_
2LS140	HSDGIFTDSY_10_SRYRKQLAVK_20_KYLAAVL-Ser(Glc)-Ser(Glc)-CONH_2_

**TABLE 2 |:** HPLC and MS data for PACAP glycopeptides.

Compound	Exact mass (Calc.)	Experimentally observed mass (ESI)	HPLC retention time (min.)
2LS72-1	3145.6495	630.1381 [M+5H] 787.4212 [M+4H] 1049.5586 [M+3H]	11.06
2LS72-2	3127.693	[M+5H]5^+^ 626.59, [M+4H]4^+^ 782.95, [M+3H]3^+^ 1043.57, [M+2H]2^+^ 1564.84	16.29^a^
2LS72-3	3127.693		16.33^a^
2LS80Gluc	3376.778	[M+5H]5^+^ 676.40, [M+4H]4^+^ 845.24, [M+3H]3^+^ 1126.64, [M+2H]2^+^ 1689.42	10.50
			16.26^a^
2LS80Lact	3538.831	[M+5H]5^+^ 708.81, [M+4H]4^+^ 885.77, [M+3H]3^+^ 1180.66, [M+2H]2^+^ 1770.36	15.66^a^
2LS80Cel	3538.831	[M+5H]5^+^ 708.84, [M+4H]4^+^ 885.74, [M+3H]3^+^ 1180.67, [M+2H]2^+^ 1770.46	10.53
2LS80Mel	3538.831	[M+5H]5^+^ 708.84, [M+4H]4^+^ 885.82, [M+3H]3^+^ 1180.74, [M+2H]2^+^ 1770.52	10.32
CRA3000	3362.76	561.80 (M+6H)6+; 673.96 (M+5H)5+; (842.20 (M+4)4+; 1122.60 (M+3H)3+	14.637
CRA3001	3249.68	651.18 (M+5H)5+; 813.73 (M+4H)4+; 1084.62 (M+3H)3+; 1625.40 (M+2H)2+	12.989
CRA3002	3263.69	653.99 (M+5H)5+; 817.26 (M+4H)4+; 1089.65 (M+3H)3+; 1638.45 (M+2H)2+	13.28
CRA3003	3332.75	667.81 (M+5H)5+; 834.52 (M+4H)4+; 1112.67 (M+3H)3+; 1668.45 (M+2H)2+	15.251
CRA3004	3219.67	645.41 (M+5H)5+; 806.26 (M+4H)4+; 1075.64 (M+3H)3+; 1610.43 (M+2H)2+	13.048
CRA3005	3233.68	653.99 (M+5H)5+; 817.26 (M+4H)4+; 1089.65 (M+3H)3+; 1638.45 (M+2H)2+	12.931
2LS98Mel	3538.8303	[M+5H]5^+^ 708.84, [M+4H]4^+^ 885.82, [M+3H]3^+^ 1180.74, [M+2H]2^+^ 1770.52	10.32
2LS98Cell	3546.8810	[M+5H]5^+^ 710.48, [M+4H]4^+^ 887.83, [M+3H]3^+^ 1183.41, [M+2H]2^+^ 1774.53	10.56
2LS98Lac	3554.9312	[M+5H]5^+^ 712.24, [M+4H]4^+^ 889.85, [M+3H]3^+^ 1186.08, [M+2H]2^+^ 1778.59	10.71
CRA3006	3390.79	3394.52^b^	15.256
CRA3007	3376.78	676.57 (M+5H)5+; 845.92 (M+4H)4+; 1126.87 (M+3H)3+; 1689.80 (M+2H)2+	14.749
CRA3008	3376.78	676.55 (M+5H)5+; 845.43 (M+4H)4+	14.747
CRA3009	3404.81	682.16 (M+5H)5+; 852.69 (M+4H)4+; 1136.23 (M+3H)3+; 1703.92 (M+2H)2+	14.819
CRA3010	3376.78	3381.510 (MALDI)	15.165
CRA3011	3390.79	679.54 (M+5H)5+; 849.19 (M+4H)4+; 1131.88 (M+3H)3+; 1697.84 (M+2H)2+	14.877
CRA3012	3348.75	671.14 (M+5H)5+; 838.33 (M+4H)4+; 1117.56 (M+3H)3+; 1675.38 (M+2H)2+	14.203
CRA3013	3362.76	673.76 (M+5H)5+; 841.95 (M+4H)4+; 1122.25 (M+3H)3+; 1682.90 (M+2H)2+	14.413
CRA3014	3320.72	665.54 (M+5H)5+; 831.68 (M+4H)4+; 1108.23 (M+3H)3+; 1662.35 (M+2H)2+	14.067
CRA3015	3362.76	673.76 (M+5H)5+; 841.95 (M+4H)4+; 1122.58 (M+3H)3+; 1682.90 (M+2H)2+	14.296
CRA3016	3348.75	670.96 (M+5H)5+; 838.45 (M+4H)4+; 1117.60 (M+3H)3+; 1676.38 (M+2H)2+	14.285
CRA3017	3362.76	673.76 (M+5H)5+; 842.20 (M+4H)4+; 1122.24 (M+3H)3+; 1682.87 (M+2H)2+	14.496
2LS132	3538.83	591.20 (M+6H)6+; 709.13 (M+5H)5+; 886.17 (M+4H)4+	15.229
Ac-2LS132	3580.84	3584.369^b^	15.229
Ac-CRA3000	3404.77	682.1647 (M+5H)5+; 852.4536 (M+4H)4+	15.277
CRA3000-Lac	3524.82	588.6446 (M+6H)6+; 706.1725 (M+5H)5+; 882.7138 (M+4H)4+; 1176.2819 (M+3H)3+	14.899
Ac-CRA3000-Lac	3566.83	595.8141 (M+6H)6+; 714.7751 (M+5H)5+; 893.2168 (M+4H)4+; 1190.6205 (M+3H)3+	15.139
CRA3018	3376.78	563.9718 (M+6H)6+; 676.5649 (M+5H)5+; 845.4541 (M+4H)4+; 1126.9363 (M+3H)3+	15.019
Ac-CRA3018	3418.79	570.9735 (M+6H)6+; 684.9670 (M+5H)5+; 855.9565 (M+4H)4+	15.307
CRA3019	3362.76	561.6357 (M+6H)6+; 673.7615 (M+5H)5+; 841.9500 (M+4H)4+; 1122.2640 (M+3H)3+	15.176
Ac-CRA3019	3404.77	568.6375 (M+6H)6+; 682.1635 (M+5H)5+; 852.4524 (M+4H)4+; 1136.2673 (M+3H)3+	15.427
CRA3020	3348.75	670.9677 (M+5H)5+; 838.4450 (M+4H)4+; 1117.5916 (M+3H)3+	14.917
Ac-CRA3020	3390.76	679.152 (M+5H)5+; 848.9486 (M+4H)4+	15.216
CRA3021	3376.78	676.5653 (M+5H)5+; 845.4545 (M+4H)4+; 1126.9366 (M+3H)3+	15.032
Ac-CRA3021	3418.82	684.9673 (M+5H)5+; 855.9571 (M+4H)4+; 1141.2739 (M+3H)3+	15.349
2ls140	3625.8628	[M+5H]5^+^ 726.18, [M+4H]4^+^ 907.46	10.94

a 30 min HPLC, gradient.

b MALDI was used instead of ESI.

**TABLE 3 |:** cAMP stimulation of PACAP glycopeptide analogues.

	PAC1		VPAC1		VPAC2	
			
Drug	EC_50_ (nM)	E_max_ (%)	EC_50_ (nM)	E_max_ (%)	EC_50_ (nM)	E_max_ (%)
1st series						

PACAP_1–27_ (Control)	25.3 ± 7.9	100	—	—	—	—
2LS72–1	86.7 ± 43.0	81.0 ± 11.0	—	—	—	—
2LS72–2	90.7	74.3 ± 9.7	—	—	—	—
2LS72–3	48.6 ± 32.0	95.3 ± 8.1	—	—	—	—
2LS80Gluc	33.6 ± 24.0	86.3 ± 36.5	—	—	—	—
2LS80Lact	33.6 ± 24.0	143.6 ± 20.7	—	—	—	—

2nd Series						

PACAP_1–27_	0.4, 0.13, 0.34	100	14.8 ± 1.6	100	0.35 ± 0.16	100
2LS98Cell	0.84	92	0.52	93	55.6	91
2LS98Lac	0.72	93	0.45	101	193	100
2LS98Mel	0.57	99	0.55	102	9.4	86
CRA3000	25.5	85	1.3	90	241	104
CRA3001	54.5	86	4.8	90	654	107
CRA3002	>250	−79	5.9	93	>2500	−95
CRA3003	>250	−71	78.8	93	>2500	−42
CRA3004	NC	NC	1,366	85	NC	NC
CRA3005	NC	NC	1,623	78	NC	NC
PACAP1–27	0.27 ± 0.13	100	0.77 ± 0.32	100	10.6 ± 3.3	100
Ac-2LS132	2.8	96	2.2	98	27.2	101

3rd Series						

PACAP_1–27_	1.73 ± 0.56	100	4.49 ± 0.64	100	73.7 ± 13.5	100
CRA3006	596	78	319	103	>2,500	−79.6
CRA 3007	30	88	165	111	706	70
CRA3008	159	76	202	108	737	65
CRA 3009	412	75	767	125	>2,500	55
CRA3010	52.3	89	2.9	107	36.5	112
CRA3011	784	68	34.2	91	223	99
CRA 3012	81.7	80	214	118	1,332	107
CRA3013	88.9	74	20.9	113	650	88
CRA 3014	205	79	132	122	2655	122
CRA3015	448	72	120	104	866	92
CRA 3016	25.4	93	28.6	109	291	105
CRA3017	10.6	95	4.8	94	189	109

4th Series						

PACAP_1–27_ (Control) ± SEM (n = 5)	0.74 ± 0.24	100	0.98 ± 0.23	100	37.5 ± 7.9	100
CRA 3000-Lac	27.2	92	17.1	96	290	116
Ac-CRA3000	112	90	79.5	94	92.8	106
Ac-CRA 3000-Lac	21.4	86	22.1	99	42.8	114
CRA3018	998	85	998	104	>2,500	112
Ac-CRA 3018	>2,500	(35)	415	36	>2,500	(31)
CRA3019	847	65	649	72	>2,500	74
Ac-CRA 3019	1,639	58	NC	18	>2,500	62
CRA3020	>2,500	97	607	78	>10,000	Ambiguous
Ac-CRA 3020	>2,500	61	244	36	>2,500	71
CRA3021	32.3	90	37.7	103	517	74
Ac-CRA3021	110	96	105	111	151	93
2LS140	2.23	102	3.06	108	156	106

**TABLE 4 |:** Receptor selectivity ratios of PACAP glycopeptides.

Compound	VPAC1/PAC1	VPAC2/PAC1	VPAC2/VPAC1
2LS98Cell	0.62	66.19	106.92
2LS98Lac	0.63	268.06	428.89
2LS98Mel	0.96	16.49	17.09
CRA3000	0.05	9.45	185.38
CRA3001	0.09	12.00	136.25
CRA3002	—	—	—
CRA3003	—	—	—
CRA3004	—	—	—
CRA3005	—	—	—
AC2LS132	0.79	9.71	12.36
CRA3006	0.54	—	—
CRA 3007	5.50	23.53	4.28
CRA3008	1.27	4.64	3.65
CRA 3009	1.86	—	—
CRA3010	0.06	0.70	12.59
CRA3011	0.04	0.28	6.52
CRA 3012	2.62	16.30	6.22
CRA3013	0.24	7.31	31.10
CRA 3014	0.64	12.95	20.11
CRA3015	0.27	1.93	7.22
CRA 3016	1.13	11.46	10.17
CRA3017	0.45	17.83	39.38
CRA 3000-Lac	0.63	10.66	16.96
Ac-CRA3000	0.71	0.83	1.17
Ac-CRA 3000-Lac	1.03	2.00	1.94
CRA3018	1.00	—	—
Ac-CRA 3018	—	—	—
CRA3019	0.77	—	—
Ac-CRA 3019	—	—	—
CRA3020	—	—	—
Ac-CRA 3020	—	—	—
CRA3021	1.17	16.01	13.71
Ac-CRA3021	0.95	1.37	1.44
2LS140	1.37	69.96	50.98

## Footnotes

1 Dhanasekaran, M.; Palian, M.M.; Alves, I.; et al. Glycopeptides related to β-endorphin adopt helical amphipathic conformations in the presence of lipid bilayers. *J. Am. Chem. Soc*. 127, 5435–5448 (2004). https://doi.org/10.1124/jpet.104.069393

2 Hashimoto, H.; Shintani, N.; Baba, A. New Insights into the Central PACAPergic System from the Phenotypes in PACAP- and PACAP Receptor-Knockout Mice. *Ann. N.Y. Acad. Sci*. 1,070, 75–89 (2006). http://doi.org/10.1196/annals.1317.038

3 Congreve, M.; de Graaf, C.; Swain, N.A.; Tate, C.G. Impact of GPCR Structures on Drug Discovery. *Cell* 181, 81–91 (2020). https://doi.org/10.1016/j.cell.2020.03.003

4 Ramos-Alvarez, I.; Mantey, A.A.; Nakamura, T. et al. A structure-function study of PACAP using conformationally restricted analogs: Identification of PAC1 receptor-selective PACAP agonists. *Peptides* 66, 26–42 (2015). https://doi.org/10.1016/j.peptides.2015.01.009
